# Modeling conditional dependencies between recidivism and cognitive emotion regulation strategies among prisoners using a Bayesian network with interpretable summary indexes

**DOI:** 10.1371/journal.pone.0352880

**Published:** 2026-08-06

**Authors:** Younyoung Choi, Gyeongcheol Cho

**Affiliations:** 1 Department of Psychology, Ajou University, Suwon, Republic of Korea; 2 Department of Psychology, The Ohio State University, Columbus, Ohio, United States of America; University of the West Indies at Saint Augustine, TRINIDAD AND TOBAGO

## Abstract

Emotion regulation is widely recognized as a critical factor in offender rehabilitation and recidivism reduction. However, empirical research has not yet clarified how distinct patterns of cognitive emotion regulation strategies (CERS) relate to recidivism across different types of criminal offenses. We therefore constructed a Bayesian network to model the probabilistic relationships between CERS and recidivism, conditioning on crime type. To obtain a practical, parsimonious network, we first derived interpretable summary indexes from multiple CERS indicators using convex generalized structured component analysis and used these indexes as network nodes. The resulting network suggested that CERS may vary across crime types and are differentially associated with the conditional probability of recidivism. These findings highlight the potential value of considering both individual CERS profiles and crime type in future research on correctional assessment and intervention planning.

## Introduction

Reducing recidivism is a central goal in correctional psychology, as repeated offending perpetuates cycles of crime, victimization, and substantial social and economic costs [1,2]. Negative emotional states such as anger, frustration, hopelessness, and depression are known to heighten reoffending risk by impairing impulse control and decision-making [3,4]. General Strain Theory explains these emotions as responses to chronic stressors and threats [5], conditions intensified in prisons where loss of freedom and limited resources are pervasive [6,7]. Because such states are dynamic and amenable to intervention, cognitive emotion regulation strategies (CERS) have emerged as promising, clinically modifiable mechanisms linked to antisocial and violent behavior [8,9]. Effective use of CERS has been identified as crucial for lowering reoffending risk [10].

Nonetheless, it remains largely unclear how distinct patterns of CERS relate to recidivism across crime types, leaving a critical gap in the literature. Addressing this gap is particularly important in correctional contexts, where negative states are intensified and where tools that align CERS profiles with offense categories could inform more targeted and effective rehabilitation programs—hence the present study.

CERS refers to thought processes or cognitive strategies used to manage and regulate emotions during or after stressful events [11,12]. Individuals lacking effective CERS are more likely to become overwhelmed by negative emotions, which can in turn trigger aggression and violent behavior. For instance, Kemp et al. (2017) found that difficulties in emotion regulation predicted future arrests among adolescents, linking regulatory deficits to impulsivity and criminal conduct [13]. Consistently, impaired regulation skills have been associated with aggression across populations ranging from undergraduate students to community samples, psychiatric patients, and both juvenile and adult offenders [3,8,14–17].

Garnefski, Kraaij, and Spinhoven (2001) developed the Cognitive Emotion Regulation Questionnaire (CERQ) to assess nine distinct strategies for managing emotions during or after threatening or negative events [11]. These strategies include self-blame (SB), attributing blame to oneself; other-blame (OB), attributing blame to the environment or other individuals; rumination (RM), repetitively focusing on thoughts and feelings related to the negative event; catastrophizing (CAT), magnifying the severity of the experienced situation; putting into perspective (PIP), shifting perspectives to reduce the severity of the current stressful condition through comparison with other events; positive refocusing (PRF), thinking about joyful and pleasant issues unrelated to the actual event; positive reappraisal (PRA), creating positive aspects of the event to encourage personal growth; refocus on planning (ROP), directing thought toward controlling the negative event and planning next steps; and acceptance (AP), acknowledging and accepting what has been experienced.

These nine CER strategies are commonly grouped into two categories: adaptive and maladaptive [11,18]. The first four strategies—self-blame (SB), other-blame (OB), rumination (RM), and catastrophizing (CAT)—are typically classified as maladaptive forms of emotion regulation. By contrast, the remaining five—putting into perspective (PIP), positive refocusing (PRF), positive reappraisal (PRA), refocus on planning (ROP), and acceptance (AP)—are considered adaptive. Individuals who rely on adaptive strategies generally function more effectively and show greater resilience in high-stress environments, whereas those who depend on maladaptive strategies often struggle, leading to outcomes such as aggression, violence, and depressive symptoms. Roberton et al. (2014) found that offenders using maladaptive strategies reported more extensive histories of aggression than those employing adaptive ones [8]. Similarly, Velotti et al. (2014) showed that maladaptive self-regulation in response to chronic or intense negative emotions predicted aggression and loss of control over antisocial behavior [9]. The tendency toward aggression and antisocial conduct is particularly marked among individuals who depend on maladaptive strategies, underscoring the link between CERS and violent outcomes [8,9,19].

Given these patterns, recent work in forensic and correctional psychology has emphasized interventions that enhance CERS among prisoners [3,20,21]. For example, Choi et al. (2023) reported that correctional mental healthcare increasingly incorporates CER assessment—often via the CERQ—to identify adaptive and maladaptive patterns and provide targeted training [22]. Importantly, enhancing CER skills not only reduces immediate aggression but also substantially lowers the likelihood of future offending, highlighting CER as a protective factor against recidivism [23].

However, the connection between CERS and recidivism may vary across crime types, and the precise nature of these differences has not been fully explored. Because the effectiveness of rehabilitation depends, in part, on offender characteristics, there is a pressing need for studies that disentangle these associations [24]. Cultural variation adds an additional layer of interpretation. In societies influenced by East Asian cultural norms, including South Korea, emotional suppression tends to be more socially accepted than overt expression [25,26]. Consequently, suppression has been linked to worse affective outcomes among European Americans but not among East Asians [27]. Relatedly, within these cultural contexts, *acceptance* may overlap conceptually with suppression when it reflects passive resignation rather than mindful acknowledgment. In such cases, acceptance functions maladaptively—serving as an emotion-dampening or avoidance strategy—rather than as an adaptive coping mechanism. This culturally specific interpretation helps explain why acceptance can manifest as both adaptive and maladaptive within certain Asian samples, a pattern less commonly observed in Western frameworks [28,29]. Together, these observations underscore the need to examine how CERS relate to recidivism across crime types within incarcerated populations in non-Western Asian contexts.

### The present study

The primary objective of this study is to examine how specific patterns of cognitive emotion regulation strategies (CERS)—categorized as adaptive or maladaptive—are associated with recidivism across crime types within a South Korean prison population. Although CERS training plays a central role in correctional practice, few studies have tested whether the links between CERS and recidivism differ by crime type in non-Western contexts. To address this gap, we constructed a Bayesian network to model the probabilistic associations between prisoners’ CERS and recidivism across six crime categories—homicide, violent offenses, sexual violence offenses, property offenses, drug-related crimes, and others—within a South Korean prison population. As described in the following sections, Bayesian networks provide a useful statistical framework for examining conditional dependencies among random variables, represented as nodes, using a probabilistic graphical model. The resulting network was used to examine two crime-specific insights: (i) how recidivism probabilities vary according to CERS profiles and crime types, and (ii) how CERS patterns differ between first-time and repeat offenders. These estimates may inform future research on how CERS profiles and crime type can be jointly considered in correctional assessment and rehabilitation planning.

## Methods

### Participants

A total of 521 prisoners were selected from Pusan National Prison in South Korea classified under a moderate security level (S4). A team of four doctoral-level clinical psychologists assessed their psychiatric symptoms, including depression, anxiety, and cognitive impairment. Individuals manifesting severe cognitive impairment were subsequently excluded from the study. The survey was administered from April 1, 2020, to February 1, 2021. Prior to their survey participation, all prisoners were comprehensively briefed on the study’s objectives and monetary compensation, based on which they decided whether to participate in this survey. Once informed consent was obtained from those who agreed to participate, each participant completed the survey individually. Consent was obtained in written form, and no minors participated in the study.

A potential ethical concern related to compensating prisoners is the risk of undue influence, where the compensation might coerce participation. To mitigate this, we took several measures. First, we ensured that the compensation amount of 50,000 won (approximately US $40) was reasonable and not so large as to be coercive. Second, we emphasized that participation was entirely voluntary and that their decision to participate or abstain would neither confer benefits nor impose penalties regarding their future incarceration conditions. Third, we provided comprehensive briefings to ensure that prisoners fully understood their rights and the study’s purpose, and that their data would be completely anonymized. These measures ensured that participation was based on informed consent and voluntary choice, minimizing any potential ethical issues related to compensation.

Ultimately, the data from 500 inmates were included in the final analysis. Ethical approval for this study was secured from the Human Subjects Review Committee at Donga University (Ethical Approval Code: 2–1040709-AB-N-01–202001-BR-003–04).

The demographic information of the participants is presented in Table 1. The participants’ ages ranged from 20 to 60 years (Mean = 46.75, Standard Deviation = 11.52), and the mean age at first conviction was 30.22 years (SD = 12.81). Based on their most recent offense, participants were classified into six crime types: homicide (29.8%), violent offenses (15.4%), sexual violence offenses (30.0%), drug-related crimes (4.0%), property offenses (17.2%), or others (3.6%). All six crime-type categories were included in the subsequent analyses. The number of incarcerations varies among the participants (see Table 1). Regarding education levels, 17.2% had completed primary school (Year 6), 25.8% completed middle school, 44.0% had finished senior high school, and 13.0% completed tertiary or higher education. In terms of job status before incarceration, 14% of participants had a full-time job, 29% had a part-time job, and 57% were unemployed. The psychological states of the participants were assessed using several measures, including the Beck Depression Inventory (BDI: Mean = 5.50, SD = 4.92), the State-Trait Anxiety Inventory (STAI-X: Mean = 42.36, SD = 10.20), the Aggression Questionnaire (AQ: Mean = 52.68, SD = 16.06), and the Barratt Impulsiveness Scale (BIS: Mean = 46.61, SD = 10.84).

**Table 1:** Demographic information and psychological characteristics of the participants.

Variable		Mean	SD
Age		46.75	11.52
Age at first conviction		30.22	12.81
Number of incarcerations		2.98	2.63
Psychological States	BDI	5.50	4.92
	STAI-X	42.36	10.20
	AQ	52.68	16.06
	BIS	46.61	10.84
**Variable**	**Categories**	**N**	**%**
Crime type	Homicide	149	29.8
	Violent Offenses	77	15.4
	Sexual Violence Offenses	150	30.0
	Property Offenses	86	17.2
	Drug-Related Crimes	20	4.0
	Others	18	3.6
Education level	Below Primary School	13	2.6
	Primary School	73	14.6
	Middle School	129	25.8
	High School	220	44.0
	Above College	65	13.0
Job Status before incarceration	No Jobs	285	57.0
	Part-time Jobs	145	29.0
	Full-time Jobs	70	14.0
Total		500	100

### Measures

#### Cognitive emotion regulation questionnaire.

Cognitive emotion regulation strategies (CERS) were assessed using the Korean version of Cognitive Emotion Regulation Questionnaire (CERQ), validated by Ahn, Lee, and Joo (2013) [30]. The CERQ were originally developed by Garnefski, Kraaij, and Spinhoven (2001) [11] and its psychometric properties were further examined by Garnefski and Kraaij (2007) Formatting...[12]. The CERQ consists of 35 items, each employing a five-point Likert scale ranging from 1 (*Almost Never)* to 5 (*Almost Always*). It measures nine CER strategies, including self-blame (SB), other-blame (OB), rumination (RM), catastrophizing (CAT), putting into perspective (PIP), positive refocusing (PRF), positive reappraisal (PRA), refocus on planning (ROP), and acceptance (AP). In this study, the total Cronbach’s α of the K-CERQ was 0.87, and the Cronbach’s α for the sub-domains were as follows: 0.80 for SB, 0.64 for OB, 0.66 for RM, 0.75 for CAT, 0.74 for PIP, 0.60 for PRF, 0.78 for PRA, 0.74 for ROP, and 0.77 for AP.

#### Recidivism.

Recidivism was quantified based on the number of incarcerations per prisoner. The variable was encoded as a dummy variable: if a prisoner had been incarcerated more than once, the recidivism variable was coded as 2; otherwise, it was coded as 1.

### Analytic procedure

In the present analysis, we constructed a Bayesian network to model conditional dependencies among prisoners’ CERS, crime type, and recidivism. Bayesian Networks are useful statistical tools for investigating conditional dependencies among variables based on Bayes’ theorem [31,32]. Once trained, a Bayesian network can be used to derive not only the conditional probability distribution of a child node (i.e., an outcome variable) given the values of its parent nodes (i.e., explanatory variables), but also the conditional probability distribution of parent nodes given the state of a child node [33]. This dual capability distinguishes Bayesian Networks from traditional regression-based approaches, such as logistic regression, which typically estimate a unidirectional conditional relationship from one fitted model. In the present context, this feature of Bayesian Networks allowed us to examine both the conditional probability distribution of recidivism given prisoners’ CERS components and crime type, and the probabilistic profiles of CERS conditional on recidivism status and crime type.

We used a discrete Bayesian network, in which each node is represented by a finite set of states and the dependencies among nodes are summarized through conditional probability tables. Although Bayesian network extensions can accommodate continuous variables, we proceeded with the traditional discrete Bayesian network framework because the substantive aim of the study was to model interpretable categorical patterns of cognitive emotion regulation and recidivism across crime types. In correctional assessment and intervention planning, decision-making often involves categorical judgments, such as identifying whether a risk-related characteristic is low or high, or whether a specific intervention target is more or less salient for a given offender group. A discrete Bayesian network is therefore well aligned with the present goal of representing conditional dependency patterns in a form that can be directly inspected and interpreted.

Despite these advantages, discrete Bayesian Networks can be susceptible to a high-dimensional setting, in which the number of cases is not sufficiently large compared to the number of nodes and states in the model [34]. This issue is particularly relevant when multiple CERS indicators are modeled jointly with crime type and recidivism in a correctional sample. To address this issue, we followed the two-stage procedure proposed by Cho et al. (2025) [34]. In this procedure, Convex Generalized Structured Component Analysis (Convex GSCA) is first used to derive low-dimensional, interpretable composite indexes (i.e., components) representing CERS [35]. These composite indexes are then used as nodes in the Bayesian network to construct a more parsimonious model.

**Fig 1:**
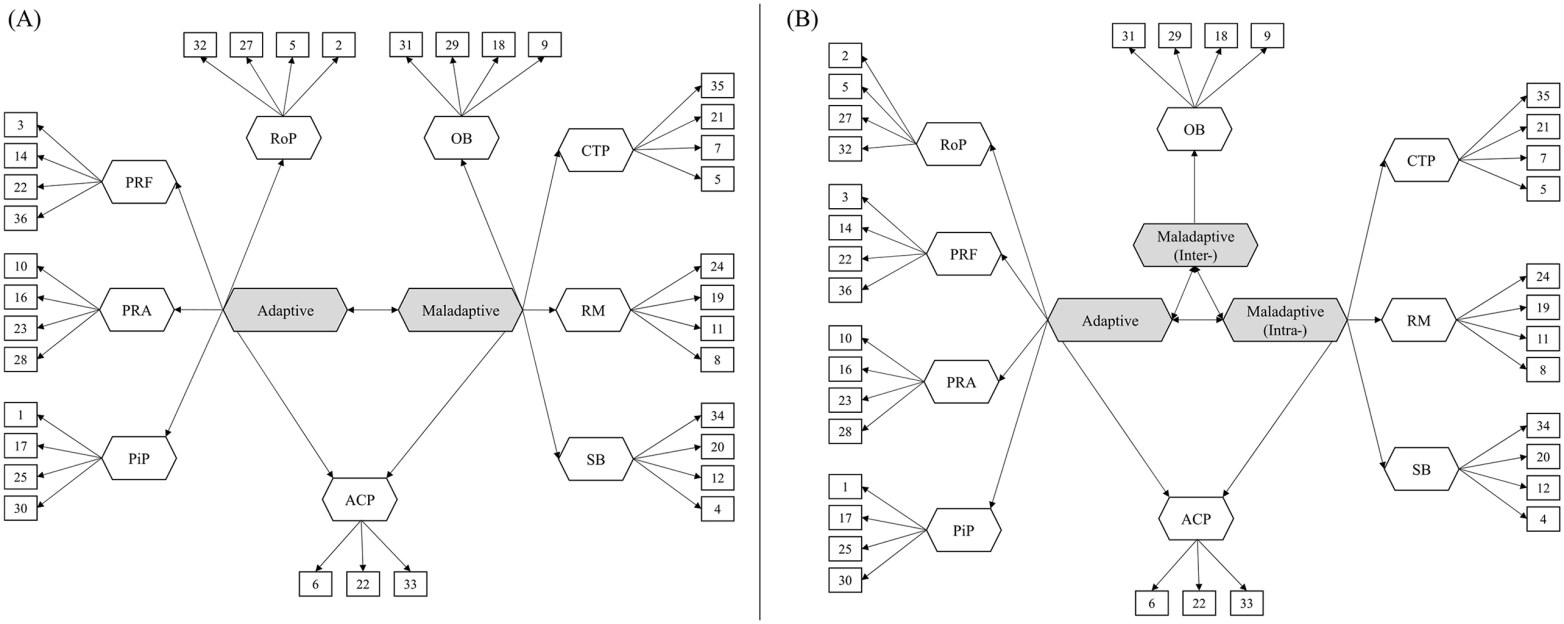
Two competing GSCA models. Squares denote items, hexagons signify components, directional arrows indicate loadings, and bi-directional arrows represent correlations. The second-order components are colored in gray. For simplicity, weights and error terms are omitted (RoP = Refocus on planning, PRA = positive reappraisal, PiP = putting into perspective, ACP = acceptance, SB = self-blame, RM = rumination, CTP = catastrophizing, and OB = other-blame). Panel (A) shows the model with two second-order components, whereas Panel (B) shows the model with three second-order components.

Specifically, considering several validity studies conducted on the CERQ scale [11,30,36], we considered the two competing GSCA models with second-order components, as shown in Fig 1. Both models commonly combine the 35 items into nine first-order components or composite indexes, each of which represents a specific cognitive coping strategy. The directional arrows extending from a component to an item block, called loading parameters, indicate the component serves as a summary index for the corresponding item block.

In addition, both GSCA models define second-order components as weighted sums of the nine first-order components, which represent more general cognitive strategies (e.g., adaptive and maladaptive strategies) that encompass the nine localized ones. As depicted by directional arrows between components of two levels, these second-order components are expected to explain their corresponding first-order component block. The adaptive strategy comprises putting into perspective (PIP), positive refocusing (PRF), positive reappraisal (PRA), and refocus on planning (ROP), whereas the maladaptive strategy includes self-blame (SB), other-blame (OB), rumination (RM), and catastrophizing (CAT). Unlike previous studies [11,18], we treated acceptance (AP) as both an adaptive and a maladaptive indicator because some AP items may reflect resignation/abandonment (e.g., “I cannot change anything about it”), which may be particularly relevant in East Asian cultural contexts that emphasize harmony, endurance, and emotional restraint [37,38].

The difference between the two GSCA models is that Model B additionally splits the maladaptive strategy into intrapersonal and interpersonal ones, categorizing SB, RM, and CAT as the former, and OB as the latter. As their name implies, the intrapersonal strategy focuses on one’s own feelings, thoughts, and experiences, whereas the interpersonal strategy is associated with directing thoughts, feelings, and blame towards others. This specification in Model B is based on the reports in previous studies that indicate OB may covary only weakly, or not at all, with the other maladaptive strategies [30,36].

We fitted the two GSCA models to the dataset of 500 cases via GSCA Prime [39] and compared their goodness-of-fit values. We used 10^−5^ as the tolerance level and 100 as the maximum number of iterations for the parameter estimation algorithm. For model evaluation, we used FIT for dependent variables (FIT^UD^) and out-of-bag prediction errors for dependent variables (OPE^UD^) [35,40]. FIT^UD^ indicates the (weighted) average proportion of variance in the dependent variables, including items and first-order components, explained by the model. Its value corresponds to the (weighted) average R^2^ and therefore can be used to evaluate the explanatory power of the model. On the other hand, OPE^D^ can be used for model comparison, as it assesses the predictive generalizability of the competing GSCA models. Lower OPE^D^ values indicate better predictive performance for the dependent variables, including indicators and first-order components, relative to the competing model. In addition, we tested the statistical significance of weight and loading parameter estimates. If all the weight and loading estimates between a component and its corresponding item block (or a second-order component and its first-order component block) are statistically significant, it indicates that every item within the block contributes to forming the component and the component subsequently explains its item block as their summary index. We used the percentile bootstrap method [41,42] to calculate the standard errors (SEs) and 95% confidence intervals (CIs) of the weight and loading estimates.

After selecting the better-fitting GSCA model in Stage 1, we used the resulting components as nodes in the Bayesian network [32]. Their scores were then used to estimate the Bayesian network parameters in Stage 2. Because a discrete Bayesian network requires categorical node states, the GSCA-derived CERS component scores were discretized into three theoretically interpretable frequency-based states before parameter estimation; the specific cutoffs are reported with the Bayesian network results.

We used Netica 6.08 to construct the Bayesian network and applied the EM algorithm to estimate its conditional probability tables [43]. We then evaluated the trained network using both an in-sample AUC and a cross-validated AUC. The AUC summarizes the discriminative ability of the network, with.5 corresponding to chance-level discrimination and larger values indicating greater discrimination [44]. The in-sample AUC was treated as a descriptive index of apparent discriminative performance within the analytic sample, whereas the cross-validated AUC was used to examine the extent to which this discriminative pattern was retained for held-out cases.

Finally, we used the fitted Bayesian network to calculate various conditional probabilities. For example, we examined the probability of being incarcerated once conditional on a given crime type and CERS profiles, as well as the probability of specific CERS profiles conditional on recidivism status and crime type.

## Results

### Stage 1: GSCA

The two GSCA models commonly have FIT^UD^ = .568 for the 35 items, indicating that the first-order CERS components in both models explain 56.8% of the total variance of all the items. Their OPE^UD^ value is smaller than 1 (OPE^UD^ = .435), indicating that their prediction error for the items is expected to be smaller than that of the null model. In contrast, the two models (A and B) have different FIT^UD^ and OPE^UD^ values for the eight first-order CERS components. Specifically, Model A has a FIT^UD^ of.557 for the first-order components while Model B has a FIT^UD^ of.645 for the same components, indicating that Models A and B explain 55.7% and 64.5%, respectively, of the total variance of the first-order components. Model B, therefore, explains an additional 8.8% of the variance of the first-order components compared to Model A. Also, the OPE^UD^ value of Model B (.359) is lower than that of Model A (.449), suggesting that Model B is expected to yield a smaller prediction error for the first-order components than Model A across samples. These results support the division of the maladaptive strategy into two sub-categories (i.e., intrapersonal and interpersonal), as shown in Model B, and the creation of distinct composite indexes for each sub-category.

The results of the statistical testing for individual parameter estimates lead us to the same conclusion. Table 2 presents the weight and loading estimates of the two GSCA models, along with their standard errors and 95% confidence intervals (CI), along with correlation estimates between components and their indicators. Overall, all the weight and loading estimates between first-order components and items were statistically significant, with most correlation estimates exceeding.6. In Model A, however, the weight and loading estimates between the maladaptive strategy and other-blame components was not statistically significant. It indicates that the other-blame component in Model A does not contribute to forming the maladaptive strategy, and consequently, the maladaptive strategy fails to explain the variance of the other-blame component as its summary index. This issue is resolved in Model B, where a second-order component is assigned separately to the other-blame component. Therefore, we selected Model B as the final GSCA model, based on which we generated low-dimensional data for CERS components for the next-stage analysis.

**Table 2:** Results for the two GSCA models.

Model	Component	Indicator	Weights				Loading				*r*
			Estimate	SE	95% CI		Estimate	SE	95% CI		
A & B	Refocus on planning	2	0.26	0.01	[0.23, 0.28]		1.02	0.05	[0.92, 1.12]		0.78
	Refocus on planning	15	0.25	0.01	[0.22, 0.27]		0.98	0.05	[0.88, 1.08]		0.78
	Refocus on planning	27	0.24	0.01	[0.22, 0.27]		0.97	0.05	[0.86, 1.06]		0.78
	Refocus on planning	32	0.26	0.01	[0.23, 0.28]		1.03	0.05	[0.92, 1.12]		0.74
	Positive refocusing	3	0.25	0.01	[0.22, 0.28]		1.00	0.05	[0.88, 1.10]		0.73
	Positive refocusing	14	0.21	0.01	[0.18, 0.24]		0.84	0.06	[0.72, 0.95]		0.69
	Positive refocusing	22	0.26	0.01	[0.24, 0.28]		1.03	0.04	[0.94, 1.10]		0.77
	Positive refocusing	36	0.28	0.01	[0.26, 0.30]		1.09	0.03	[1.03, 1.16]		0.84
	Positive reappraisal	10	0.19	0.02	[0.16, 0.22]		0.75	0.07	[0.60, 0.88]		0.61
	Positive reappraisal	16	0.24	0.01	[0.22, 0.26]		0.94	0.04	[0.85, 1.02]		0.75
	Positive reappraisal	23	0.29	0.01	[0.26, 0.31]		1.12	0.04	[1.05, 1.19]		0.82
	Positive reappraisal	28	0.28	0.01	[0.26, 0.30]		1.09	0.04	[1.01, 1.16]		0.80
	Putting into perspective	1	0.19	0.03	[0.14, 0.24]		0.77	0.11	[0.52, 0.96]		0.53
	Putting into perspective	17	0.26	0.02	[0.22, 0.29]		1.02	0.07	[0.86, 1.14]		0.69
	Putting into perspective	25	0.26	0.02	[0.22, 0.30]		1.03	0.07	[0.87, 1.16]		0.68
	Putting into perspective	30	0.28	0.02	[0.25, 0.32]		1.12	0.06	[0.99, 1.21]		0.77
	Acceptance	6	0.35	0.02	[0.30, 0.40]		1.04	0.07	[0.90, 1.16]		0.75
	Acceptance	26	0.37	0.02	[0.34, 0.40]		1.09	0.04	[1.00, 1.16]		0.83
	Acceptance	33	0.28	0.02	[0.24, 0.32]		0.83	0.07	[0.68, 0.94]		0.70
	Self-blame	4	0.24	0.01	[0.22, 0.26]		0.93	0.05	[0.83, 1.01]		0.75
	Self-blame	12	0.29	0.01	[0.28, 0.31]		1.12	0.02	[1.07, 1.16]		0.89
	Self-blame	20	0.29	0.01	[0.28, 0.31]		1.13	0.03	[1.07, 1.19]		0.86
	Self-blame	34	0.17	0.01	[0.15, 0.20]		0.67	0.06	[0.56, 0.78]		0.63
	Rumination	8	0.25	0.01	[0.23, 0.27]		0.99	0.04	[0.91, 1.06]		0.79
	Rumination	11	0.23	0.01	[0.21, 0.25]		0.92	0.04	[0.84, 0.99]		0.74
	Rumination	19	0.25	0.01	[0.23, 0.27]		1.00	0.03	[0.93, 1.06]		0.82
	Rumination	24	0.27	0.01	[0.25, 0.29]		1.08	0.03	[1.01, 1.14]		0.79
	Catastrophizing	5	0.21	0.02	[0.17, 0.24]		0.82	0.08	[0.65, 0.96]		0.63
	Catastrophizing	7	0.31	0.01	[0.28, 0.33]		1.20	0.04	[1.11, 1.27]		0.83
	Catastrophizing	21	0.25	0.02	[0.21, 0.28]		0.97	0.07	[0.83, 1.09]		0.69
	Catastrophizing	35	0.24	0.02	[0.20, 0.27]		0.94	0.07	[0.78, 1.06]		0.66
	Other-blame	9	0.27	0.01	[0.25, 0.29]		1.05	0.04	[0.97, 1.12]		0.80
	Other-blame	18	0.27	0.01	[0.25, 0.29]		1.06	0.03	[1.00, 1.12]		0.82
	Other-blame	29	0.19	0.02	[0.16, 0.22]		0.75	0.08	[0.60, 0.89]		0.56
	Other-blame	31	0.27	0.01	[0.25, 0.29]		1.06	0.03	[0.99, 1.12]		0.81
A	Adaptive	Refocus on planning	0.19	0.01	[0.17, 0.21]		0.87	0.05	[0.76, 0.95]		0.73
	Adaptive	Positive refocusing	0.21	0.01	[0.19, 0.24]		0.95	0.05	[0.84, 1.05]		0.70
	Adaptive	Positive reappraisal	0.28	0.01	[0.26, 0.31]		1.25	0.03	[1.18, 1.31]		0.88
	Adaptive	Putting into perspective	0.21	0.01	[0.19, 0.23]		0.92	0.04	[0.83, 1.00]		0.77
	Adaptive	Acceptance	0.11	0.02	[0.06, 0.15]		0.64	0.09	[0.45, 0.83]		0.48
	Maladaptive (Intra-)	Acceptance	0.16	0.04	[0.09, 0.24]		0.48	0.08	[0.31, 0.65]		0.43
	Maladaptive (Intra-)	Self-blame	0.31	0.03	[0.26, 0.36]		1.05	0.04	[0.97, 1.11]		0.81
	Maladaptive (Intra-)	Rumination	0.36	0.02	[0.32, 0.39]		1.07	0.09	[0.88, 1.23]		0.81
	Maladaptive (Intra-)	Catastrophizing	0.22	0.02	[0.19, 0.25]		0.82	0.06	[0.68, 0.94]		0.77
	Maladaptive (Intra-)	Other-blame	−0.05	0.04	[−0.13, 0.03]		−0.09	0.11	[−0.29, 0.14]		−0.08
B	Adaptive	Refocus on planning	0.19	0.01	[0.17, 0.21]		0.87	0.05	[0.77, 0.96]		0.73
	Adaptive	Positive refocusing	0.21	0.01	[0.18, 0.24]		0.95	0.05	[0.84, 1.05]		0.70
	Adaptive	Positive reappraisal	0.28	0.01	[0.26, 0.31]		1.25	0.03	[1.18, 1.31]		0.88
	Adaptive	Putting into perspective	0.21	0.01	[0.19, 0.23]		0.92	0.04	[0.84, 1.00]		0.77
	Adaptive	Acceptance	0.11	0.02	[0.07, 0.15]		0.65	0.10	[0.45, 0.83]		0.48
	Maladaptive (Intra-)	Acceptance	0.15	0.03	[0.09, 0.22]		0.49	0.09	[0.33, 0.68]		0.41
	Maladaptive (Intra-)	Self-blame	0.30	0.02	[0.26, 0.33]		1.08	0.04	[0.99, 1.16]		0.80
	Maladaptive (Intra-)	Rumination	0.34	0.02	[0.31, 0.38]		1.14	0.04	[1.05, 1.22]		0.82
	Maladaptive (Intra-)	Catastrophizing	0.21	0.02	[0.18, 0.24]		0.87	0.04	[0.79, 0.94]		0.78
	Maladaptive (Inter-)	Other-blame	1.00	0.00	[1.00, 1.00]		1.00	0.00	[1.00, 1.00]		1.00

*Note*. This table provides the weight and loading estimates of the two GSCA models and their standard errors (SE) and 95% confidence intervals (CI), along with correlation estimates between components and their indicator.

### Stage 2: Bayesian Networks

**Fig 2:**
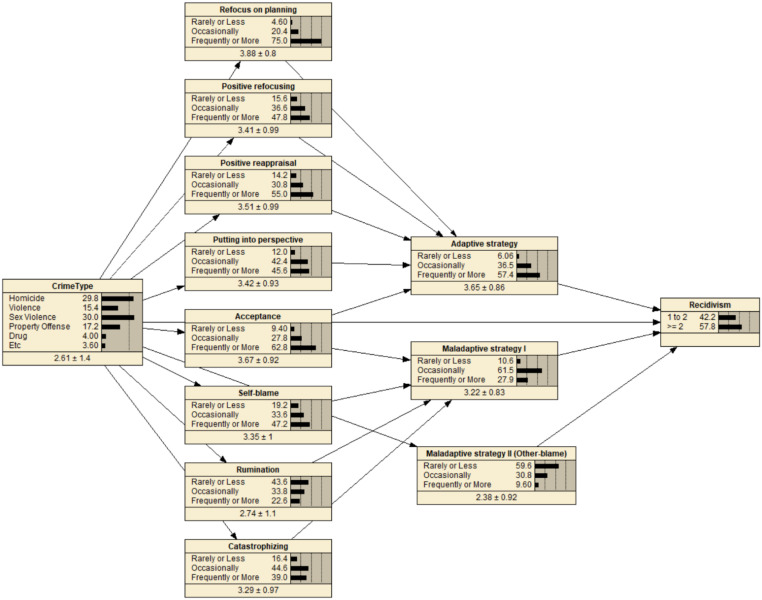
Bayesian network for cognitive emotion regulation strategies and Recidivism.

Fig 2 depicts the Bayesian network constructed with first- and second-order CERS components derived from Stage 1. The first-order components correspond to nine CERS, while the second-order components comprise one adaptive strategy and two maladaptive strategies. Since the second maladaptive strategy is identical to the other-blame strategy, we included only a single node (i.e., box) to represent both components. Each first- and second-order CERS component was discretized into three states using the following cutoffs: 1–2.5 = ‘Rarely or less,’ 2.5–3.5 = ‘Occasionally,’ and 3.5–5 = ‘Frequently or more.’ This discretization was possible because the component scores for the CERQ strategies obtained from Convex GSCA can be interpreted in relation to the original five-point response scale, ranging from 1 (“Almost Never”) to 5 (“Almost Always”) [35]. This allowed us to use theoretically interpretable substantive thresholds rather than purely distribution-based cut-points, such as tertiles based on the sample distribution. The arrows connecting the first-level components to the second-level components illustrate how the distributions of the latter are determined by the former. Likewise, the arrows leading from the second-order components and crime types to recidivism indicate that these variables exert influence on recidivism. Notably, crime types and recidivism were also included in the network.

**Fig 3:**
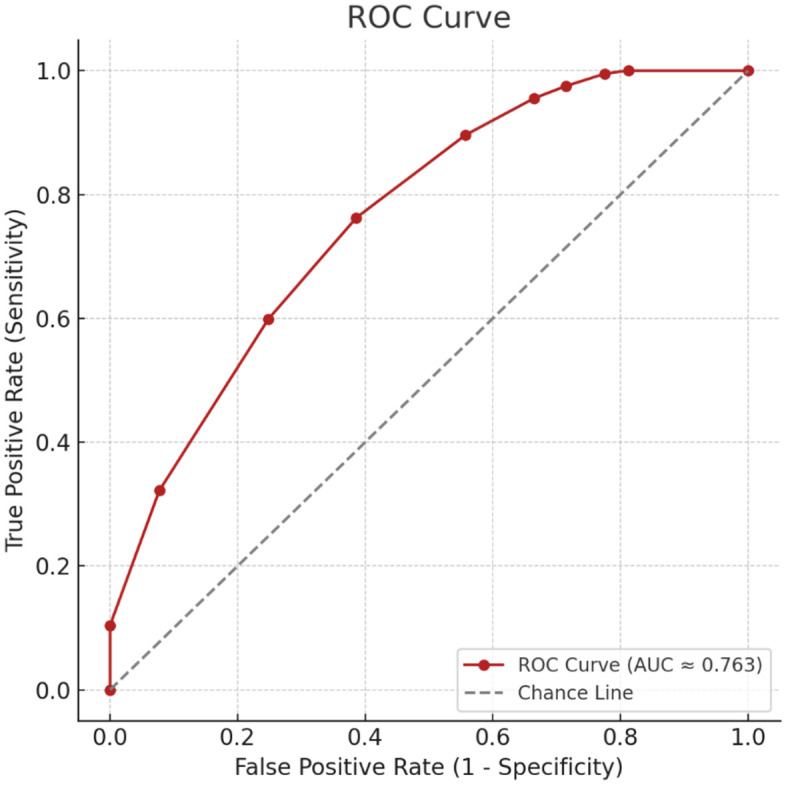
ROC curve of the Bayesian network.

The in-sample ROC curve of the Bayesian network is displayed in Fig 3. The in-sample AUC was.763, indicating that the fitted network showed apparent discriminative performance within the analytic sample. However, because this value was computed using the same data used for parameter estimation, it should not be interpreted as evidence of out-of-sample predictive generalizability. To examine the extent to which this discriminative pattern was retained for held-out cases, we additionally computed a leave-one-out cross-validated AUC (LOOCV-AUC). The LOOCV-AUC was.552, indicating limited out-of-sample discriminative performance. Thus, the fitted Bayesian network was used primarily to examine conditional probability patterns linking prisoners’ CERS components, crime type, and recidivism, rather than as a validated high-accuracy prediction model.

**Fig 4:**
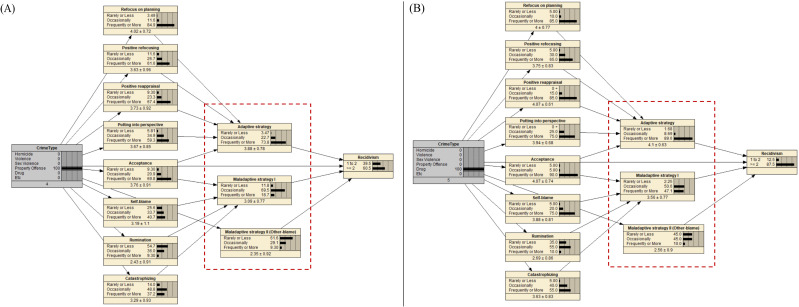
Example conditional probability patterns of CERS across criminal categories in the Bayesian network. (A) Conditional probability pattern for property offenders. (B) Conditional probability pattern for drug offenders.

Fig 4 illustrates how the Bayesian network can be used to descriptively compare posterior CERS probability patterns across crime categories. As an example, property offenders and drug offenders appear to show different probability distributions across CERS strategies, with the drug-offender subgroup displaying a descriptively higher probability of maladaptive strategies.

**Fig 5:**
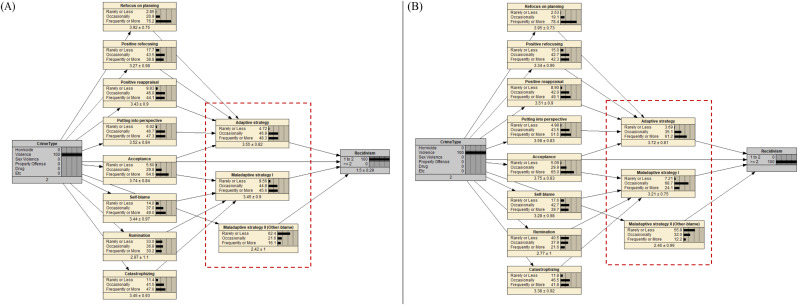
Conditional probability patterns of adaptive and maladaptive CERS among violent offenders by recidivism status. (A) Conditional probability pattern for first-time offenders. (B) Conditional probability pattern for re-offenders.

In addition, the Bayesian network can be used to descriptively examine differences in CERS patterns within specific offender groups according to recidivism status. For example, Fig 5 illustrates posterior CERS probability patterns for first-time offenders and re-offenders within the violent-offender group. Focusing on the “frequently or more” response category, the probability of this category for Adaptive Strategy was 61.2% among violent re-offenders, compared to 48.3% among first-time violent offenders. Conversely, the probability of the “frequently or more” category for Maladaptive Strategy I was 24.1% among re-offenders, compared with 45.6% among first-time offenders. These descriptive patterns suggest that, within the violent-offender group in the present sample, re-offenders showed a higher probability of being in the “Frequently or more” state for Adaptive Strategy, whereas first-time offenders showed a higher probability of being in the “Frequently or more” state for Maladaptive Strategy I.

**Fig 6:**
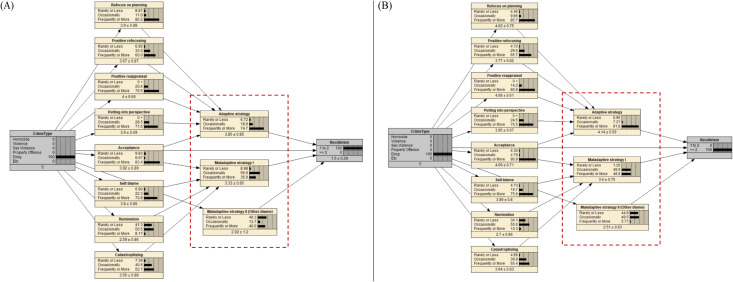
Conditional probability patterns of adaptive and maladaptive CERS among drug offenders by recidivism status. (A) Conditional probability pattern for first-time offenders. (B) Conditional probability pattern for re-offenders.

As another illustrative example, Fig 6 illustrates posterior CERS patterns among drug offenders. Within the drug-offender group, re-offenders showed a descriptively higher probability of being in the “Frequently or more” state for Adaptive Strategy (91.8% vs. 74.7%) and Maladaptive Strategy I (48.8% vs. 35.0%) than first-time offenders. In contrast, re-offenders showed a lower probability of being in the “Frequently or more” state for Maladaptive Strategy II (other-blame; 5.7% vs. 40.0%).

**Fig 7:**
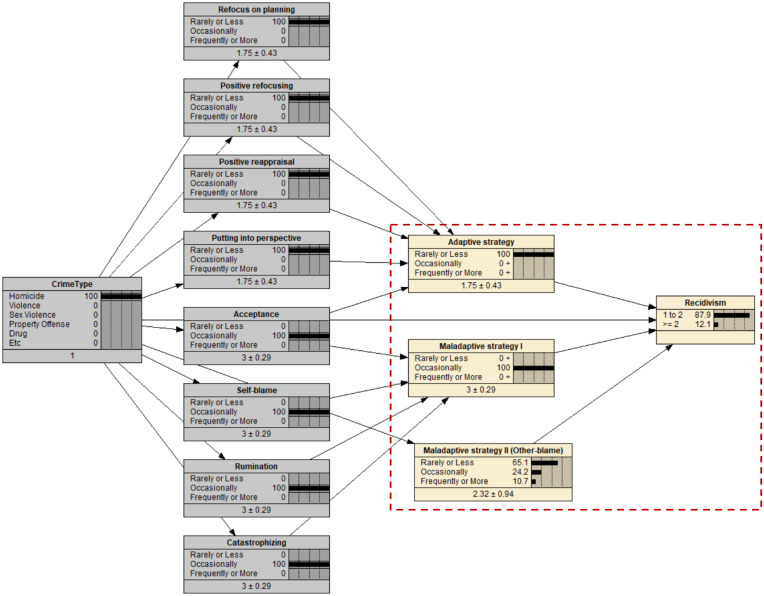
Examples of conditional probability of recidivism in a Bayesian network.

Lastly, Fig 7 illustrates how the fitted Bayesian network can be used to compute posterior probabilities at the recidivism node given a specified crime type and CERS profile. In this illustrative profile, the specified profile represented a homicide offender with rare or less frequent use of Adaptive Strategy and occasional use of Maladaptive Strategy I. Given these inputs, the fitted network produced a posterior probability of reoffending of 87.9%. This example illustrates the network’s ability to generate conditional probabilistic summaries for specified offender profiles.

Taken together, these results suggest that the Bayesian network provided an interpretable and descriptive representation of conditional dependencies between CERS components and recidivism across crime types in the analytic sample. However, given the modest cross-validated AUC and the small size of some crime-type subgroups, particularly drug offenders, the posterior probability patterns for subgroups and specified offender profiles should be interpreted cautiously and should not be taken as evidence of validated predictive performance or individualized risk prediction.

## Discussion

### Summary and Implications

This study applied Cho et al.’s (2025) [34] two-stage approach to build a parsimonious Bayesian network modeling the conditional dependencies between cognitive emotion regulation strategies (CERS) and recidivism across crime types. This framework contributes to understanding complex interrelations among these variables by providing a graphical representation that facilitates intuitive interpretation. Conventional statistical methods often focus on group-level patterns, such as mean differences or correlations, and may be less suited for representing the probabilistic nature of the associations between CERS and recidivism. In contrast, the Bayesian network developed in this study offers a probabilistically grounded framework that supports the examination of conditional probability patterns at both group and profile levels while accounting for the interdependencies inherent in psychological and behavioral processes. The findings from the Bayesian network can be summarized in three specific facets.

At the group level, the Bayesian network enabled the examination of differences in CERS probability patterns across offender types. Fig 4 descriptively compared drug and property offenders and showed possible differences in posterior CERS probability profiles. These patterns suggest that CERS may not be uniformly distributed across offender groups and may reflect offense-type-related differences in CERS profiles. This observation points to the potential value of considering crime type when identifying emotion-regulation targets for correctional intervention. For instance, the present results raise the possibility that emotion-regulation targets may differ between drug and property offenders.

This group-level insight was further elaborated in Figs 5 and 6, which examined within-group differences in CERS probability patterns by recidivism status. In the present sample, violent re-offenders showed a higher posterior probability of being in the “Frequently or more” state for Adaptive Strategy, whereas drug re-offenders showed higher posterior probabilities of being in the “Frequently or more” state for both Adaptive Strategy and Maladaptive Strategy I.

The pattern in which violent re-offenders showed a higher posterior probability of frequent Adaptive Strategy use can be cautiously interpreted through the lens of instrumental aggression [45] and moral disengagement [46,47]. These cognitive mechanisms are not inherently prosocial; they may serve as mental justifications—such as “it wasn’t that bad” or “they had it coming”—or as planning-oriented cognitive processes that may facilitate goal-directed behavior without necessarily reducing violent behavior. Thus, a high probability of Adaptive Strategy use in this context may not necessarily indicate prosocial or violence-inhibiting functioning, because such strategies may be used to rationalize or organize behavior rather than to inhibit it [48].

In contrast, the finding that drug re-offenders showed high use of both adaptive and maladaptive strategies may be considered in relation to the concept of emotion-regulation flexibility deficits [49]. One possible interpretation is that these individuals may possess multiple CER tools but fail to select the appropriate one for a given situation or to switch strategies when one proves ineffective. Under stress or craving, rumination and self-blame can overwhelm the benefits of reappraisal or planning, resulting in active yet potentially ineffective coping, which may be relevant to relapse and recidivism risk. This interpretation aligns with meta-analytic evidence [50] linking ER strategies to psychopathology, including substance-related problems, and with theoretical perspectives emphasizing that flexibility, rather than frequency, of strategy use predicts psychological adjustment. Taken together, these patterns suggest that the function and impact of CERS on recidivism may differ by crime type.

Beyond group-level insights, the Bayesian network also illustrates how conditional probability queries can be generated for specified offender profiles. As shown in Fig 7, when an offender profile is specified by crime type and CERS response pattern, the fitted network can propagate this information and compute the corresponding posterior probability at the recidivism node. This feature demonstrates the potential usefulness of Bayesian networks for summarizing how combinations of crime type and CERS patterns are probabilistically linked to recidivism within the fitted model. In addition, the network can support simple scenario-based queries by comparing how different specified CERS profiles are associated with posterior probabilities of recidivism.

### Limitations and future studies

Despite its contributions, this study has several limitations that should be considered in future research. First, because the variables were measured through self-report measures, potential biases such as social desirability and subjective misperception may have influenced participants’ responses. Future research could benefit from incorporating objective indicators—such as institutional records, physiological responses, or observational data—to obtain a more comprehensive assessment of cognitive emotion regulation strategies.

Second, although a sample size of more than 500 is substantial given the specialized nature of the offender population, some offense-type subgroups were small, particularly the drug offender subgroup. In addition, because the discrete Bayesian network involved multiple parent-state configurations, some conditional probability estimates may have been based on sparse cells. This issue was also reflected in the modest leave-one-out cross-validated AUC, suggesting that the predictive generalizability of the current network should be interpreted cautiously. Future studies with larger and more balanced samples across crime types are needed to evaluate the stability and generalizability of the observed CERS–recidivism dependency patterns.

Third, our findings are based on a specific population, which may limit generalizability of the conditional dependency patterns observed among CERS, recidivism, and crime type. For instance, in this study, *acceptance* (AP) was used to represent both adaptive and maladaptive strategies to reflect the collectivist, harmony-oriented norms and face-saving tendencies that have been documented in East Asian societies, including South Korea [29,51]. This decision aligns with cross-cultural work on interdependent self-construals and context-sensitive attribution in these cultural contexts, as well as scholarship on *face* and related social expectations [37,52]. In such contexts, acceptance may overlap conceptually with emotional suppression when it reflects passive resignation rather than mindful acknowledgment, functioning maladaptively as an avoidance or emotion-dampening strategy. However, this culturally embedded interpretation may not generalize uniformly across cultural contexts, and future studies should examine whether the role of acceptance in the CERS structure is replicated in other populations.

Fourth, another potential limitation lies in the susceptibility of discrete Bayesian networks to model complexity. Although discrete Bayesian networks are useful for representing interpretable categorical dependency patterns, the number of conditional probability table entries increases rapidly as the number of parent nodes and the number of states per parent node increases, which can make model fitting challenging. This issue is particularly relevant in the present study because CERS components, crime type, and recidivism were modeled jointly in a sample with some small crime-type subgroups.

To manage this complexity, we first derived low-dimensional CERS components through Convex GSCA and used theoretically interpretable three-state discretization thresholds for their scores. These decisions allowed us to specify a parsimonious Bayesian network structure with a limited number of parent nodes for recidivism. However, as reflected in the modest LOOCV-AUC value, the model’s predictive generalizability remained limited, and the resulting conditional probability patterns may be sensitive to the number of states and the selected thresholds. Alternative discretization schemes, a larger number of states, or more data-driven cut-points may yield different conditional probability patterns and potentially improve predictive generalizability. Future research should therefore examine the robustness of the BN results across different state definitions, thresholding strategies, and model structures using larger samples.

Fifth, uncertainty around the conditional probability estimates in the Bayesian network was not directly evaluated in the present study. Because the component scores used to construct the Bayesian network were estimated from GSCA in Stage 1, the stability of the conditional probability estimates may be affected not only by sampling variability in the Bayesian network estimation but also by uncertainty in the Stage 1 component-score estimation. Future research should evaluate the stability of the conditional probability patterns across the full two-stage pipeline, for example through resampling-based procedures that repeat both component-score estimation and Bayesian network estimation.

Lastly, while the current study employed a cross-sectional design, future work could explore longitudinal extensions using Dynamic Bayesian Networks [53,54] to examine temporal changes in emotion regulation and subsequent recidivism outcomes. In particular, following currently incarcerated individuals after release and examining whether their CERS profiles predict recidivism over a defined follow-up period would provide stronger temporal evidence than the present cross-sectional analysis. Such longitudinal data would allow future studies to move beyond descriptive conditional dependency patterns and more rigorously evaluate the dynamic and potentially causal relationships between emotion regulation and recidivism.

Despite these limitations, the present study contributes to correctional psychology by demonstrating how a two-stage Bayesian network approach can be used to model conditional dependency patterns between CERS components, crime type, and recidivism in an incarcerated sample. Rather than serving as a validated individualized prediction tool, the fitted network provides an interpretable and probabilistic representation of how CERS profiles and recidivism status are linked across crime types. These results may inform future research on how emotion-regulation targets can be conceptualized in correctional assessment and rehabilitation planning. To promote transparency and further research, we have made the final Bayesian network publicly available at at this link, allowing readers to inspect the network structure, node states, and conditional probability tables.
