# Supplementary material for: Modeling conditional dependencies between recidivism and cognitive emotion regulation strategies among prisoners using a Bayesian network with interpretable summary indexes
Source: PLoS One. 2026 Aug 6;21(8):e0352880. doi: 10.1371/journal.pone.0352880 (PMC13446662; doi:10.1371/journal.pone.0352880)
Supplement: Supplementary file 3 [file pone.0352880.s003.docx]

**PONE-D-25-57503: Modeling conditional dependencies between recidivism and cognitive emotion regulation strategies among prisoners using a Bayesian network with interpretable summary indexes**

We thank the Editor and two anonymous reviewers for their thoughtful and constructive comments. For their convenience, we repeat every comment in italics and then respond to the comment in regular font.

**Response to Reviewer #1**

1. *The manuscript makes a valuable contribution to correctional psychology by demonstrating how Bayesian networks can model complex, probabilistic relationships between emotion regulation and recidivism in ways that conventional statistical methods cannot. The findings have both theoretical implications for understanding offense-specific emotion regulation patterns and practical applications for personalized intervention planning. The authors have thoroughly addressed previous reviewer concerns, and the manuscript is suitable for publication in PLOS ONE.* *The authors have thoroughly addressed previous reviewer concerns, and the manuscript is suitable for publication in PLOS ONE.*

-- We sincerely appreciate the reviewer’s positive assessment of our manuscript and the thoughtful comments they provided in the previous review. We made substantial efforts to address their concerns, and we are very pleased to hear that the reviewer appreciate both our revisions and the manuscript’s contribution.

**Response to Reviewer #2**

**Major comments**

1. *[Discretization of GSCA Component Scores Not Described] Netica requires discrete nodes, yet the continuous GSCA component scores from Stage 1 are entered into the BN without any description of how they were discretized. The Methods section must specify: (1) the discretization method (equal-frequency, equal-width, percentile, or substantive thresholds); and (2) the number of discrete states per node. The Results section refers to "higher," "medium," and "lower" levels (p. 22), implying a three-state scheme, but this is never stated or justified. The Discussion should also acknowledge that discretization entails information loss and that results are sensitive to the choice of cut-points. Future research directions worth noting include sensitivity analyses comparing alternative discretization schemes, or the use of continuous BN extensions (e.g., conditional Gaussian models) that eliminate the need for discretization entirely.*

-- We appreciate the reviewer’s helpful comment. Although the Bayesian network file included the discrete states and cut-points, we agree that the discretization procedure should have been described and justified more explicitly in the manuscript. Accordingly, we revised the Methods and Results sections to specify the discretization procedure for the CERS component scores and its rationale.

In the Methods section, we added the following clarification:

“After selecting the better-fitting GSCA model in Stage 1, we used the resulting components as nodes in the Bayesian network [32]. Their scores were then used to estimate the Bayesian network parameters in Stage 2. Because a discrete Bayesian network requires categorical node states, the GSCA-derived CERS component scores were discretized into three theoretically interpretable frequency-based states before parameter estimation; the specific cutoffs are reported with the Bayesian network results.” (p. 12)

In the Results section, we further specified the number of states, the cutoffs, and the rationale for using substantive thresholds:

“Figure 2 depicts the Bayesian network constructed with first- and second-order CERS components derived from Stage 1. The first-order components correspond to nine CERS, while the second-order components comprise one adaptive strategy and two maladaptive strategies. Since the second maladaptive strategy is identical to the other-blame strategy, we included only a single node (i.e., box) to represent both components. Each first- and second-order CERS component was discretized into three states using the following cutoffs: 1–2.5 = ‘Rarely or less,’ 2.5–3.5 = ‘Occasionally,’ and 3.5–5 = ‘Frequently or more.’ This discretization was possible because the component scores for the CERQ strategies obtained from Convex GSCA can be interpreted in relation to the original five-point response scale, ranging from 1 (“Almost Never”) to 5 (“Almost Always”) [35]. This allowed us to use theoretically interpretable substantive thresholds rather than purely distribution-based cut-points, such as tertiles based on the sample distribution.” (p. 15)

We also revised the terminology to use the frequency-based state labels consistently throughout the manuscript. In addition, we updated the Discussion section to acknowledge that discretization may involve some loss of information and that the Bayesian network results can be sensitive to the number of states and selected cut-points. We added the following limitation and future research direction:

“… However, as reflected in the modest LOOCV-AUC value, the model’s predictive generalizability remained limited, and the resulting conditional probability patterns may be sensitive to the number of states and the selected thresholds. Alternative discretization schemes, a larger number of states, or more data-driven cut-points may yield different conditional probability patterns and potentially improve predictive generalizability. Future research should therefore examine the robustness of the BN results across different state definitions, thresholding strategies, and model structures using larger samples.” (p. 22)

Lastly, we appreciate the reviewer’s suggestion regarding continuous Bayesian network extensions. Nonetheless, we retained the discrete Bayesian network framework because it provides readily interpretable conditional probability tables that are well aligned with the substantive and applied goals of the study. Accordingly, we clarified in the Methods section why the conventional discrete Bayesian network framework was appropriate for the present study:

“We used a discrete Bayesian network, in which each node is represented by a finite set of states and the dependencies among nodes are summarized through conditional probability tables. Although Bayesian network extensions can accommodate continuous variables, we proceeded with the traditional discrete Bayesian network framework because the substantive aim of the study was to model interpretable categorical patterns of cognitive emotion regulation and recidivism across crime types. In correctional assessment and intervention planning, decision-making often involves categorical judgments, such as identifying whether a risk-related characteristic is low or high, or whether a specific intervention target is more or less salient for a given offender group. A discrete Bayesian network is therefore well aligned with the present goal of representing conditional dependency patterns in a form that can be directly inspected and interpreted.” (p. 10)

2. *[Model Evaluation: AUC Computed on Training Data]* The reported AUC of .763 appears to be evaluated on the same 500-case dataset used for parameter estimation, producing an optimistically biased performance estimate — a concern compounded by the relatively small sample and the number of nodes in the network. The authors should replace this in-sample AUC with a proper out-of-sample estimate via k-fold cross-validation (e.g., 10-fold, reporting mean AUC with confidence intervals), a held-out test set, or bootstrapped bias-corrected estimation. The current claim that the network "outperforms random guessing" is insufficiently supported without one of these approaches.

-- We appreciate the reviewer’s important comment. We agree that the AUC reported in the previous version was computed on the same data used for parameter estimation and therefore should not be interpreted as an out-of-sample estimate of predictive performance. We also agree that the previous statement that the network “outperforms random guessing” could be read as overstating the predictive performance of the model, and we have removed this statement from the revised manuscript.

At the same time, we would like to clarify that the primary aim of the present study was not to develop or validate a high-accuracy recidivism prediction model. As reflected in the title and abstract, the purpose of the study was to model conditional dependencies between recidivism and cognitive emotion regulation strategies among prisoners, conditioning on crime type, using a Bayesian network with interpretable summary indexes. Thus, the originally reported in-sample AUC was intended as a descriptive index of the fitted network’s apparent discriminative performance within the analytic sample, rather than as evidence of predictive generalizability.

To address the reviewer’s concern, we first updated the Methods section to describe the two AUC values and their interpretations as follows:

“... We then evaluated the trained network using both an in-sample AUC and a cross-validated AUC. The AUC summarizes the discriminative ability of the network, with .5 corresponding to chance-level discrimination and larger values indicating greater discrimination [44]. The in-sample AUC was treated as a descriptive index of apparent discriminative performance within the analytic sample, whereas the cross-validated AUC was used to examine the extent to which this discriminative pattern was retained for held-out cases.” (p. 13)

We additionally computed a leave-one-out cross-validated AUC and reported its value in the Results section. Because the Bayesian network structure was fixed rather than learned from the data, each leave-one-out iteration re-estimated the conditional probability tables using the remaining $N-1$cases and then obtained the predicted probability of recidivism for the held-out case. The final LOOCV-AUC was computed from the held-out predicted probabilities across all cases.

The resulting LOOCV-AUC was 0.552, indicating limited out-of-sample discriminative performance and a substantial reduction from the in-sample AUC. Accordingly, we revised the Results and Discussion sections to clarify the limited predictive generalizability of the fitted Bayesian network and to reposition the model as an exploratory and interpretable representation of conditional dependency patterns, rather than as a validated high-accuracy prediction model. For example, in the Results section, we now state:

“... To examine the extent to which this discriminative pattern was retained for held-out cases, we additionally computed a leave-one-out cross-validated AUC (LOOCV-AUC). The LOOCV-AUC was .552, indicating limited out-of-sample discriminative performance. Thus, the fitted Bayesian network was used primarily to examine conditional probability patterns linking prisoners’ CERS components, crime type, and recidivism, rather than as a validated high-accuracy prediction model.” (p. 16)

We also revised the interpretation of subgroup- and profile-level posterior probabilities throughout the manuscript to avoid implying validated individualized prediction. In particular, we now summarize the Results section as follows:

“Taken together, these results suggest that the Bayesian network provided an interpretable and descriptive representation of conditional dependencies between CERS components and recidivism across crime types in the analytic sample. However, given the modest cross-validated AUC and the small size of some crime-type subgroups, particularly drug offenders, the posterior probability patterns for subgroups and specified offender profiles should be interpreted cautiously and should not be taken as evidence of validated predictive performance or individualized risk prediction.” (p. 18)

Finally, we explicitly acknowledged this issue as a limitation in the Discussion section. Specifically, we added the following statement:

“... In addition, because the discrete Bayesian network involved multiple parent-state configurations, some conditional probability estimates may have been based on sparse cells. This issue was also reflected in the modest leave-one-out cross-validated AUC, suggesting that the predictive generalizability of the current network should be interpreted cautiously. Future studies with larger and more balanced samples across crime types are needed to evaluate the stability and generalizability of the observed CERS–recidivism dependency patterns.” (p. 21)

3. *[Critical Citation Error: Korean CERQ Reference] In the Measures section (p. 8), the authors write: “Cognitive-emotion regulation strategies (CERS) were assessed using the Korean version of Cognitive Emotion Regulation Questionnaires (CERQ), validated by Ahn, Lee, and Joo (2013) [43].” However, Reference [43] in the reference list is:
Pavlenko T, Rosen D von. Bayesian network classifiers in a high dimensional framework. AI '02… (2002).
The correct reference for Ahn, Lee, and Joo (2013) appears to be [47] in the reference list. This is a substantive citation error that misattributes the validation of the primary measurement instrument. The authors must verify that all in-text citation numbers match their reference list entries.*

-- We thank the reviewer for catching this important citation error. The error appears to have occurred when the reference numbering changed during a previous revision of the manuscript. We corrected the Measures section so that the Korean validation of the CERQ is now cited as Ahn, Lee, and Joo (2013) [30]. We also clarified the original CERQ references by citing Garnefski, Kraaij, and Spinhoven (2001) [11] for the original development of the CERQ and Garnefski and Kraaij (2007) [12] for further psychometric evidence.

In addition, we carefully checked the in-text citations and reference list throughout the manuscript and corrected the mismatches we identified. We appreciate the reviewer’s careful reading and helpful attention to this issue.

4. *[Drug Offender Subgroup Too Small for Reliable Inference] The drug-related crimes subgroup comprises only 20 participants (4.0% of the sample). Despite this, Figure 6 and the associated Discussion present specific conditional probability estimates for drug offenders to two decimal places (e.g., “91.8% vs. 74.7%” for adaptive strategy use). Conditional probability tables for a subgroup of n=20, further divided by recidivism status, are estimated from single-digit cell counts, rendering these estimates highly unstable and their interpretation unreliable.
The authors must either: (a) acknowledge this as a critical limitation and refrain from drawing substantive conclusions from the drug-offender analysis; or (b) provide bootstrapped confidence intervals for all conditional probabilities reported in Figures 5–7 to reflect the uncertainty associated with small cell sizes. Presenting point estimates without uncertainty quantification for such small subgroups risks misleading readers.*

-- We appreciate the reviewer’s important comment. We agree that the drug-related crime subgroup was small and that conditional probability estimates for this subgroup may be unstable, especially when further conditioned on recidivism status and CERS component levels. Because correctional datasets of this kind are difficult to obtain, our intention in retaining the drug-related crime category was to provide a descriptive account of the crime types represented in the available sample, not to draw strong subgroup-specific substantive or clinical conclusions. In the revised manuscript, we therefore rewrote the relevant Results and Discussion passages so that the drug-offender findings are presented as sample-based, descriptive, and exploratory. For example, we now summarize the findings as follows:

“Taken together, these results suggest that the Bayesian network provided an interpretable and descriptive representation of conditional dependencies between CERS components and recidivism across crime types in the analytic sample. However, given the modest cross-validated AUC and the small size of some crime-type subgroups, particularly drug offenders, the posterior probability patterns for subgroups and specified offender profiles should be interpreted cautiously and should not be taken as evidence of validated predictive performance or individualized risk prediction.” (p. 18)

We also explicitly acknowledged this issue in the Limitations and Future Studies section:

“Although a sample size of more than 500 is substantial given the specialized nature of the offender population, some crime-type subgroups were small, particularly the drug offender subgroup. In addition, because the discrete Bayesian network involved multiple parent-state configurations, some conditional probability estimates may have been based on sparse cells. This issue was also reflected in the modest leave-one-out cross-validated AUC, suggesting that the predictive generalizability of the current network should be interpreted cautiously. Future studies with larger and more balanced samples across crime types are needed to evaluate the stability and generalizability of the observed CERS–recidivism dependency patterns.” (p. 21)

**Minor Comments**

1. *[Descriptive Statistics Inconsistency (Text vs. Table 1)] The manuscript text (p. 8) reports the mean number of incarcerations as 2.39 (SD = 2.20), but Table 1 reports the same variable as Mean = 2.98, SD = 2.63. These values are irreconcilable. The authors must verify which figure is correct and ensure consistency between the text and the table. Additionally, the variable “Age of first conviction” (Mean = 30.22, SD = 12.81) appears in Table 1 but is not mentioned in the text; it should either be described or removed from the table.*

-- We agree with the reviewer. The value reported in the main text was a typographical error. We verified the descriptive statistics and corrected the text to be consistent with Table 1. We also revised the text to describe the age of first conviction, which was included in Table 1 but not previously mentioned in the manuscript.

2. *[Disposition of “Others” Offense Category] Table 1 includes an “Others” offense category (n = 18, 3.6%). It is unclear whether these participants were included in or excluded from the Bayesian network analysis, which models five specific crime types (homicide, violent offenses, sexual violence, property, drug). If excluded, the effective analysis sample would be n = 482, not n = 500. This must be clarified.*

-- We appreciate the reviewer’s comment. In the revised manuscript, we clarified that the “Others” crime category (*n* = 18) was not excluded from the analysis. Rather, all six crime-type categories were retained in the subsequent analyses, and the effective analysis sample remained *N* = 500. Specifically, we added the following clarification:

“Based on their most recent offense, participants were classified into six crime types: homicide (29.8%), violent offenses (15.4%), sexual violence offenses (30.0%), drug-related crimes (4.0%), property offenses (17.2%), or others (3.6%). All six crime-type categories were included in the subsequent analyses.” (p. 8)

3. [Uncertainty Propagation Across Stages] The two-stage procedure treats GSCA-derived component scores as fixed observed values in the BN, thereby ignoring the estimation uncertainty inherent in Stage 1. In particular, the standard errors and confidence intervals reported in Table 2 for GSCA weight and loading estimates are not carried forward to Stage 2. This leads to underestimation of the uncertainty surrounding the conditional probability estimates produced by the BN. The authors should at minimum acknowledge this limitation and, ideally, consider a sensitivity analysis or bootstrapped approach that captures the combined uncertainty across both stages.

-- We appreciate the reviewer’s thoughtful comment. We agree that the two-stage procedure did not carry forward uncertainty from Stage 1 GSCA component-score estimation into Stage 2 Bayesian network estimation. Accordingly, we revised the Discussion section to acknowledge that uncertainty around the Bayesian network conditional probability estimates was not directly evaluated in the present study and that such uncertainty may be affected by both Bayesian network estimation and the preceding component-score estimation. We also added a future research direction suggesting that the stability of the conditional probability patterns should be evaluated across the full two-stage pipeline, for example through resampling-based procedures that repeat both component-score estimation and Bayesian network estimation.

Specifically, we added the following limitation:

“Fifth, uncertainty around the conditional probability estimates in the Bayesian network was not directly evaluated in the present study. Because the component scores used to construct the Bayesian network were estimated from GSCA in Stage 1, the stability of the conditional probability estimates may be affected not only by sampling variability in the Bayesian network estimation but also by uncertainty in the Stage 1 component-score estimation. Future research should evaluate the stability of the conditional probability patterns across the full two-stage pipeline, for example through resampling-based procedures that repeat both component-score estimation and Bayesian network estimation.” (pp. 22-23)

4. *[Typographical Errors] The running head on page 1 reads “MODELING CONDITONAL DEPENENCY B/W RECIDIVISM AND CERS,” which contains two spelling errors (“CONDITONAL” and “DEPENENCY”) and an informal abbreviation (“B/W”). The full running head on subsequent pages retains the spelling errors. These should be corrected.*

-- We thank the reviewer for noting these typographical errors. We corrected the running head throughout the revised manuscript by fixing the spelling of “conditional” and “dependency/dependencies” and removing the informal abbreviation “B/W.”

**Closing Remarks**

We thank the reviewers again for their careful and constructive comments. We believe that the revisions have substantially improved the manuscript, particularly with respect to interpretive caution, citation accuracy, methodological transparency, and acknowledgment of methodological limitations. We hope that the revised manuscript is now suitable for publication in PLOS ONE.
