# Supplementary material for: Modeling conditional dependencies between recidivism and cognitive emotion regulation strategies among prisoners using a Bayesian network with interpretable summary indexes
Source: PLoS One. 2026 Aug 6;21(8):e0352880. doi: 10.1371/journal.pone.0352880 (PMC13446662; doi:10.1371/journal.pone.0352880)
Supplement: Supplementary file 4 [file pone.0352880.s001.docx]

**PEER REVIEW REPORT**

*PLOS ONE Manuscript Review*

# *Modeling conditional dependencies between recidivism and cognitive–emotion regulation strategies among prisoners using a Bayesian network with interpretable summary indexes*

# Major Concerns

## . Discretization of GSCA Component Scores Not Described

Netica requires discrete nodes, yet the continuous GSCA component scores from Stage 1 are entered into the BN without any description of how they were discretized. The Methods section must specify: (1) the discretization method (equal-frequency, equal-width, percentile, or substantive thresholds); and (2) the number of discrete states per node. The Results section refers to "higher," "medium," and "lower" levels (p. 22), implying a three-state scheme, but this is never stated or justified.

The Discussion should also acknowledge that discretization entails information loss and that results are sensitive to the choice of cut-points. Future research directions worth noting include sensitivity analyses comparing alternative discretization schemes, or the use of continuous BN extensions (e.g., conditional Gaussian models) that eliminate the need for discretization entirely.

## . Model Evaluation: AUC Computed on Training Data

The reported AUC of .763 appears to be evaluated on the same 500-case dataset used for parameter estimation, producing an optimistically biased performance estimate — a concern compounded by the relatively small sample and the number of nodes in the network. The authors should replace this in-sample AUC with a proper out-of-sample estimate via k-fold cross-validation (e.g., 10-fold, reporting mean AUC with confidence intervals), a held-out test set, or bootstrapped bias-corrected estimation. The current claim that the network "outperforms random guessing" is insufficiently supported without one of these approaches.

## Critical Citation Error: Korean CERQ Reference

In the Measures section (p. 8), the authors write: “Cognitive-emotion regulation strategies (CERS) were assessed using the Korean version of Cognitive Emotion Regulation Questionnaires (CERQ), validated by Ahn, Lee, and Joo (2013) [43].” However, Reference [43] in the reference list is:

*Pavlenko T, Rosen D von. Bayesian network classifiers in a high dimensional framework. AI '02… (2002).*

The correct reference for Ahn, Lee, and Joo (2013) appears to be [47] in the reference list. This is a substantive citation error that misattributes the validation of the primary measurement instrument. **The authors must verify that all in-text citation numbers match their reference list entries.**

## Drug Offender Subgroup Too Small for Reliable Inference

The drug-related crimes subgroup comprises only 20 participants (4.0% of the sample). Despite this, Figure 6 and the associated Discussion present specific conditional probability estimates for drug offenders to two decimal places (e.g., “91.8% vs. 74.7%” for adaptive strategy use). Conditional probability tables for a subgroup of n=20, further divided by recidivism status, are estimated from single-digit cell counts, rendering these estimates highly unstable and their interpretation unreliable.

The authors must either: (a) acknowledge this as a critical limitation and refrain from drawing substantive conclusions from the drug-offender analysis; or (b) provide bootstrapped confidence intervals for all conditional probabilities reported in Figures 5–7 to reflect the uncertainty associated with small cell sizes. Presenting point estimates without uncertainty quantification for such small subgroups risks misleading readers.

# Minor Concerns

## Descriptive Statistics Inconsistency (Text vs. Table 1)

The manuscript text (p. 8) reports the mean number of incarcerations as 2.39 (SD = 2.20), but Table 1 reports the same variable as Mean = 2.98, SD = 2.63. **These values are irreconcilable.** The authors must verify which figure is correct and ensure consistency between the text and the table. Additionally, the variable “Age of first conviction” (Mean = 30.22, SD = 12.81) appears in Table 1 but is not mentioned in the text; it should either be described or removed from the table.

## Disposition of “Others” Offense Category

Table 1 includes an “Others” offense category (n = 18, 3.6%). It is unclear whether these participants were included in or excluded from the Bayesian network analysis, which models five specific crime types (homicide, violent offenses, sexual violence, property, drug). If excluded, the effective analysis sample would be n = 482, not n = 500. This must be clarified.

## Uncertainty Propagation Across Stages

The two-stage procedure treats GSCA-derived component scores as fixed observed values in the BN, thereby ignoring the estimation uncertainty inherent in Stage 1. In particular, the standard errors and confidence intervals reported in Table 2 for GSCA weight and loading estimates are not carried forward to Stage 2. This leads to underestimation of the uncertainty surrounding the conditional probability estimates produced by the BN. The authors should at minimum acknowledge this limitation and, ideally, consider a sensitivity analysis or bootstrapped approach that captures the combined uncertainty across both stages.

## Typographical Errors

The running head on page 1 reads “MODELING CONDITONAL DEPENENCY B/W RECIDIVISM AND CERS,” which contains two spelling errors (“CONDITONAL” and “DEPENENCY”) and an informal abbreviation (“B/W”). The full running head on subsequent pages retains the spelling errors. These should be corrected.
