# Supplementary material for: Modeling conditional dependencies between recidivism and cognitive emotion regulation strategies among prisoners using a Bayesian network with interpretable summary indexes
Source: PLoS One. 2026 Aug 6;21(8):e0352880. doi: 10.1371/journal.pone.0352880 (PMC13446662; doi:10.1371/journal.pone.0352880)
Supplement: Supplementary file 5 [file pone.0352880.s002.docx]

**PONE-D-23-26797: Modeling conditional dependencies between recidivism and cognitive–emotion regulation strategies among prisoners using a Bayesian network with interpretable summary indexes**

We thank the Academic Editor and anonymous reviewers for their thoughtful and constructive comments, which substantially improved the manuscript. For ease of review, we reproduce each comment in *italics* followed by our response in regular text. All revisions are highlighted in red in the annotated manuscript. Page and line references in our responses refer to the revised, line-numbered version.

**Response to Reviewer 1**

**Abstract**

1. *Consider breaking down longer sentences into shorter, more digestible ones to enhance clarity. For instance, the sentence beginning with "Following the two-stage procedure..." could be split into two.*

*--* We have reviewed the abstract thoroughly and revised its structure and sentences to improve readability based on your suggestion as follows:

“Emotion regulation is widely recognized as a critical factor in offender rehabilitation and recidivism reduction. However, empirical research has not yet clarified how distinct patterns of cognitive emotion regulation strategies (CERS) relate to recidivism across different types of criminal offenses. We therefore constructed a Bayesian network to model the probabilistic relationships between CERS and recidivism, conditioning on offense type. To obtain a practical, parsimonious network, we first derived interpretable summary indexes from multiple CERS indicators using convex generalized structured component analysis. These indexes were then used as network nodes. The resulting network suggests that CERS differ systematically across offense types and are differentially associated with the likelihood of recidivism. These findings underscore the importance of incorporating both individual CERS profiles and offense type into the design of correctional interventions aimed at reducing recidivism.” (p.1)

2. *Ensure that technical terms like "Generalized Structured Component Analysis (GSCA)" are accessible to a broader audience. A brief, simple explanation or alternative phrasing could be beneficial.*

-- We understand that this term may sound technical to a broader audience. While we cannot rephrase the term as it is the specific name of a statistical technique, we have clarified its purpose in the revised abstract as follows:

“··· To obtain a practical, parsimonious network, we first derived interpretable summary indexes from multiple CERS indicators using convex generalized structured component analysis. ···” (p.1)

**Introduction**

1. *Begin with a few sentences that set the stage for why this research is crucial. Highlight the gap in the current understanding of CER in forensic psychology and its implications for recidivism.*

-- In the revision, we began the first paragraph by emphasizing the importance of reducing recidivism within the field of forensic psychology. We then underscored the relevance of emotion regulation strategies to the prison environment and their connection to recidivism. Finally, we highlighted a critical gap in the literature—namely, that the relationships between distinct patterns of cognitive emotion regulation strategies (CERS) and recidivism across offense types have not yet been examined—and discussed its implications for understanding recidivism.

“Reducing recidivism is a central goal in correctional psychology, as repeated offending perpetuates cycles of crime, victimization, and substantial social and economic costs [1,2]. Negative emotional states such as anger, frustration, hopelessness, and depression are known to heighten reoffending risk by impairing impulse control and decision-making [3,4]. General Strain Theory explains these emotions as responses to chronic stressors and threats [5,6], conditions intensified in prisons where loss of freedom and limited resources are pervasive [7–9]. Because such states are dynamic and amenable to intervention, cognitive emotion regulation strategies (CERS) have emerged as promising, clinically modifiable mechanisms linked to antisocial and violent behavior [10–12]. Effective use of CERS has been identified as crucial for lowering reoffending risk [13,14].

Nonetheless, it remains largely unclear how distinct patterns of CERS relate to recidivism across offense types, leaving a critical gap in the literature. Addressing this gap is particularly important in correctional contexts, where negative states are intensified and where tools that align CERS profiles with offense categories could inform more targeted and effective rehabilitation programs—hence the present study.” (p.3)

2. *While the detailed literature review is informative, consider condensing it to ensure a focused narrative. Aim to integrate studies more seamlessly, highlighting key findings that directly support the need for your research.*

-- Based on your suggestion, we completely restructured and revised the Introduction by condensing the literature review to create a more coherent narrative and improve overall readability. We also revised the Present Study section accordingly. All revised portions are highlighted in red.

3. *State the primary aim of your study more distinctly. This could be placed at the beginning of the last paragraph for greater emphasis. A clear, concise statement of the study’s objectives will help readers grasp the central focus immediately.*

-- We have re-structured and revised “the present study section” in such a way that readers can grasp the main objectives of this study clearly as follows:

“The primary objective of this study is to examine how specific patterns of cognitive emotion regulation strategies (CERS)—categorized as adaptive or maladaptive—are associated with recidivism across offense types within a South Korean prison population. Although CERS training plays a central role in correctional practice, few studies have tested whether the links between CERS and recidivism differ by offense type in non-Western contexts. To address this gap, we constructed a Bayesian network to model the probabilistic associations between prisoners’ CERS and recidivism across five crime categories: homicide, violent offenses, sexual violence offenses, property offenses, and drug-related crimes. As described in the following sections, Bayesian networks provide a powerful statistical framework for examining conditional dependencies among random variables (nodes) using a probabilistic graphical model. The resulting network yielded two offense-specific insights: (i) how reoffending probabilities vary according to CERS profiles and offense types, and (ii) how CERS patterns differ between first-time and repeat offenders. These estimates can be translated into practitioner-facing tools that map individual CERS profiles to offense categories, thereby supporting tailored, context-sensitive rehabilitation planning and ultimately more effective correctional interventions.” (p.5)

4. *Expand slightly on how cultural differences could influence your study's approach or findings, particularly given the study’s South Korean context.*

-- We have added a few sentences regarding the cultural differences and the characteristics of some CER strategies in the context of Asian culture.

“… Cultural variation adds an additional layer of interpretation. In societies influenced by East Asian cultural norms, including South Korea, emotional suppression tends to be more socially accepted than overt expression [32,33]. Consequently, suppression has been linked to worse affective outcomes among European Americans but not among East Asians [34]. Relatedly, within these cultural contexts, *acceptance* may overlap conceptually with suppression when it reflects passive resignation rather than mindful acknowledgment. In such cases, acceptance functions maladaptively—serving as an emotion-dampening or avoidance strategy—rather than as an adaptive coping mechanism. This culturally specific interpretation helps explain why acceptance can manifest as both adaptive and maladaptive within certain Asian samples, a pattern less commonly observed in Western frameworks [35–37]. …” (pp.4-5)

5. *that terms like 'Bayesian Networks' and 'cognitive-emotional regulation' are clearly defined for readers who may not be familiar with them.*

-- In the revised introduction, we have added a brief description of Bayesian Networks as follows:

“… To address this gap, we constructed a Bayesian network to model the probabilistic associations between prisoners’ CERS and recidivism across five crime categories: homicide, violent offenses, sexual violence offenses, property offenses, and drug-related crimes. As described in the following sections, Bayesian networks provide a powerful statistical framework for examining conditional dependencies among random variables (nodes) using a probabilistic graphical model. …” (p.5)

In the second paragraph of the original introduction, we have provided a definition of cognitive-emotional regulation along with relevant information as follows:

“CERS refers to thought processes or cognitive strategies used to manage and regulate emotions during or after stressful events [15,16]. Individuals lacking effective CERS are more likely to become overwhelmed by negative emotions, which can in turn trigger aggression and violent behavior. For instance, Baumeister (1990) showed that heightened emotional arousal may diminish cognitive control, reducing regulation capacity and leading to aggression [17]. Similarly, Kemp et al. (2017) found that difficulties in emotion regulation predicted future arrests among adolescents, linking regulatory deficits to impulsivity and criminal conduct [18]. Consistently, impaired regulation skills have been associated with aggression across populations ranging from undergraduate students to community samples, psychiatric patients, and both juvenile and adult offenders [3,11,19–23]” (pp.2-3)

6. *Emphasize what makes your study unique. Is it the use of BNs in this context, the specific population you’re studying, or the combination of crime types and CER strategies? Make it clear why your research is a significant contribution.*

-- We have emphasized the contribution of using Bayesian networks (BNs) in our study, including how they enable the integration of crime types and cognitive emotion regulation (CER) strategies among offenders in a non-Western population, in the revised Introduction:

“…The primary objective of this study is to examine how specific patterns of cognitive emotion regulation strategies (CERS)—categorized as adaptive or maladaptive—are associated with recidivism across offense types within a South Korean prison population. Although CERS training plays a central role in correctional practice, few studies have tested whether the links between CERS and recidivism differ by offense type in non-Western contexts. To address this gap, we constructed a Bayesian network to model the probabilistic associations between prisoners’ CERS and recidivism across five crime categories—homicide, violent offenses, sexual violence offenses, property offenses, and drug-related crimes—within a South Korean prison population. …” (p.5)

We also emphasized, in the *Discussion* section, the unique aspects of our study that contribute to the field. Below is a relevant excerpt:

“This study applied Cho et al.’s (2025) [38] two-stage approach to build a parsimonious Bayesian network modeling the conditional dependencies between cognitive emotion regulation strategies (CERS) and recidivism across criminal types. This framework advances understanding of complex interrelations among these variables by providing a graphical representation that facilitates intuitive interpretation. Above all, conventional statistical methods have traditionally focused on identifying group-level patterns such as mean differences or correlations, often falling short of capturing the probabilistic nature of the associations between CERS and recidivism. In contrast, the Bayesian network developed in this study offers a probabilistically grounded framework that supports inference at both macro (group) and micro (individual) levels while accounting for the interdependencies inherent in psychological and behavioral processes. The findings derived from the constructed Bayesian network provide insight into three specific facets. …” (p.16)

**Materials and Methods**

1. *Mention any potential ethical concerns related to compensating prisoners and how these were mitigated.*

-- We have added a paragraph that explains potential ethical concerns and their mitigating measures as follows:

“…Prior to their survey participation, all prisoners were comprehensively briefed on the study's objectives and monetary compensation, based on which they decided whether to participate in this survey. Once informed consent was obtained from those who agreed to participate, each participant completed the survey individually. Consent was obtained in written form, and no minors participated in the study.

A potential ethical concern related to compensating prisoners is the risk of undue influence, where the compensation might coerce participation. To mitigate this, we took several measures. First, we ensured that the compensation amount of 50,000 won (approximately US $40) was reasonable and not so large as to be coercive. Second, we emphasized that participation was entirely voluntary and that their decision to participate or abstain would neither confer benefits nor impose penalties regarding their future incarceration conditions. Third, we provided comprehensive briefings to ensure that prisoners fully understood their rights and the study's purpose, and that their data would be completely anonymized. These measures ensured that participation was based on informed consent and voluntary choice, minimizing any potential ethical issues related to compensation.

Ultimately, the data from 500 inmates were included in the final analysis. Ethical approval for this study was secured from the Human Subjects Review Committee at Donga University (Ethical Approval Code: 2-1040709-AB-N-01-202001-BR-003-04).” (p.6)

**Results**

1. *The description of the BNs model and its application is well-articulated. Consider simplifying the explanation of how the model works for readers who might not be familiar with Bayesian statistics. Emphasize the key insights gained from this analysis.*

-- In the revision, we restructured our description of the results to be more organized and accessible for a general audience. Specifically, in the first paragraph of this section, we clarified the types of information and insights that researchers can gain from the constructed Bayesian network as follows:

“Figure 2 depicts the Bayesian network constructed with first-order and second-order CERS components derived from Stage 1. … The ROC curve of this BN is displayed in Figure 3. The area under the curve (AUC) is .763, indicating that this network outperforms the random guessing in predicting prisoner’s recidivism status. Thus, we used this Bayesian network to obtain the likelihoods of prisoners reoffending given their CERS and criminal types, or to identify distinct patterns in CERS between first-time offenders and recidivists.” (pp.13-14)

In the following paragraphs, we illustrated what information one can obtain from the Bayesian network with four examples (1) an example of group differences in CERS strategies by criminal type, (2) two examples of group differences in CERS strategies by recidivism in a certain criminal type, and (3) one example of individual differences in the likelihood of recidivism. Below are parts of description

“Figure 4 illustrates that the Bayesian network allows for a comparison of CERS posterior probability patterns that vary by criminal category.” (p.14)

“In addition, differences in CERS patterns can be identified within specific offender groups according to recidivism status. For example, Figure 5 illustrates ….” (p.14)

“Lastly, given information about an offender’s criminal type and CERS profile, the Bayesian network enables a probabilistic estimation of their likelihood of recidivism. Figure 7 illustrates…” (p.15)

2. *Terms like 'FITD' and 'OPED' should be briefly re-explained for clarity, as readers might not remember their meanings from the Method section.*

-- In the revision, we have briefly described the meanings of these terms again as follows:

“The two GSCA models commonly have FIT^UD^ = .568 for the 35 items, indicating that the first-order CERS components in both models explain 56.8% of the total variance of all the items. Their OPE^UD^ value is smaller than 1 (OPE^UD^ = .435), indicating that their prediction error for the items is expected to be smaller than that of the null model. In contrast, the two models (A and B) have different FIT^UD^ and OPE^UD^ values for the eight first-order CERS components. Specifically, Model A has a FIT^D^ of .557 for the first-order components while Model B has a FIT^D^ of .645 for the same components, indicating that Models A and B explain 55.7% and 64.5%, respectively, of the total variance of the first-order components. Model B, therefore, explains an additional 8.8% of the variance of the first-order components compared to Model A. Also, the OPE^D^ value of Model B (.359) is lower than that of Model A (.449), suggesting that Model B is expected to yield a smaller prediction error for the first-order components than Model A across samples. These results support the division of the maladaptive strategy into two sub-categories (i.e., intrapersonal and interpersonal), as shown in Model B, and the creation of distinct composite indexes for each sub-category.” (p.12)

**Discussion**

1. *Emphasize the novelty and importance of using Bayesian Networks in this context. How does this approach advance understanding in the field compared to traditional methods?*

-- We highlighted methodological advantages and theoretical contributions in correctional setting.

“This study applied Cho et al.’s (2025) [38] two-stage approach to build a parsimonious Bayesian network modeling the conditional dependencies between cognitive emotion regulation strategies (CERS) and recidivism across criminal types. This framework advances understanding of complex interrelations among these variables by providing a graphical representation that facilitates intuitive interpretation. Above all, conventional statistical methods have traditionally focused on identifying group-level patterns such as mean differences or correlations, often falling short of capturing the probabilistic nature of the associations between CERS and recidivism. In contrast, the Bayesian network developed in this study offers a probabilistically grounded framework that supports inference at both macro (group) and micro (individual) levels while accounting for the interdependencies inherent in psychological and behavioral processes. The findings derived from the constructed Bayesian network provide insight into three specific facets. …

At the group level, the Bayesian network enabled the identification of structural differences in CERS patterns across offender types. …

Beyond group-level insights, the most salient implication of the Bayesian network lies in its capacity to facilitate individual-level inference and prediction. As demonstrated in Figure 7, inputting an offender’s criminal type and CERS response profile into the Bayesian network enables the network to propagate this information and calculate a personalized posterior probability of recidivism. ….

Furthermore, Bayesian Networks allow practitioners to simulate how changes in one variable propagate through a network of interacting variables, thereby supporting scenario-based simulation. …” (pp 16-18)

2. *While you've mentioned the need for future research in different contexts, briefly discuss how cultural and institutional factors in South Korea might have influenced the findings and their applicability elsewhere.*

-- We acknowledged that the findings of the study can vary across cultures, and therefore, there is a need for further studies to include a more diverse population in the Limitation and Further Studies section.

“Third, our findings are based on a specific population, which may limit the generalizability of the conditional dependence patterns observed among CERS, recidivism, and offense type. For instance, in this study, acceptance (AP) was used to represent both adaptive and maladaptive strategies to reflect the collectivist, harmony-oriented norms and face-saving tendencies prevalent in East Asian societies, including South Korea [37,60]. This decision aligns with cross-cultural work on interdependent self-construals and context-sensitive attribution in these cultural contexts, as well as scholarship on face and related social expectations [48,61]. In such contexts, acceptance may overlap conceptually with emotional suppression when it reflects passive resignation rather than mindful acknowledgment, functioning maladaptively as an avoidance or emotion-dampening strategy. However, this culturally embedded interpretation may not be universally applicable. Future studies should therefore include larger and more culturally diverse offender populations to examine the cross-cultural validity of the present findings and test whether the structure of CERS remains stable across contexts.…” (p.19-20)

3. *Provide specific recommendations for correctional psychologists and clinicians. What concrete steps can be taken to apply your findings in real-world settings?*

-- We appreciate the reviewer’s suggestion to clarify how our findings can be applied in real-world correctional settings. In response, we have added two paragraphs in the *Implications* section outlining specific, practice-oriented recommendations for correctional psychologists and clinicians, as follows:

“Beyond group-level insights, the most salient implication of the Bayesian network lies in its capacity to facilitate individual-level inference and prediction. As demonstrated in Figure 7, inputting an offender’s criminal type and CERS response profile into the Bayesian network enables the network to propagate this information and calculate a personalized posterior probability of recidivism. This predictive functionality moves beyond generalized risk classifications, offering a dynamic, individualized approach. Such outputs can directly inform correctional decision-making processes, including parole evaluation, clinical triage, and treatment planning.

Furthermore, Bayesian Networks allow practitioners to simulate how changes in one variable propagate through a network of interacting variables, thereby supporting scenario-based simulation. For instance, practitioners can manipulate specific input variables (e.g., frequency of maladaptive strategy use) and immediately observe changes in the predicted likelihood of recidivism. This feature enables the virtual testing and comparison of multiple intervention strategies. By systematically adjusting variables such as the use of particular CER strategies, practitioners can evaluate how alternative configurations influence predicted recidivism probabilities. Such simulation-based analyses promote a data-driven approach to treatment planning, allowing intervention pathways to be optimized prior to implementation—thereby enhancing both clinical precision and resource efficiency.” (p.18)

4. *Consider discussing any methodological limitations, such as the potential impact of the study design or statistical methods used. Also, acknowledge any potential ethical concerns related to studying a prison population.*

-- We appreciate the reviewer’s thoughtful suggestion to elaborate on the methodological and ethical limitations of our study. In response, we have expanded the Limitations and Future Studies section to include detailed discussion of both the study design and the Bayesian network’s methodological constraints, as shown below:

“Despite its contributions, this study has several limitations that should be considered in future research. First, because all variables were measured through self-report instruments, potential biases such as social desirability and subjective misperception may have influenced participants’ responses. Future research could benefit from incorporating objective indicators—such as physiological measures or behavioral observations—to obtain a more comprehensive assessment of emotion regulation strategies.

Second, although a sample size of more than 500 is substantial given the specialized nature of the offender population—particularly considering the smaller size of certain subgroups such as drug offenders—future studies with larger samples could improve the generalizability of these findings.

…

Lastly, another potential limitation lies in the susceptibility of Bayesian networks to model complexity. Although BNs are highly effective in capturing probabilistic and multidimensional dependencies, the large number of possible conditional relationships and posterior probability paths can make model fitting challenging. In this study, we addressed this issue by reducing data dimensionality through convex generalized structured component analysis. However, it was necessary to restrict the number of parent nodes for the recidivism variable. Future research could build on this foundation by incorporating additional variables—such as demographic, psychosocial, or institutional factors—into Bayesian networks with larger samples to more accurately represent the multifaceted nature of offender rehabilitation. Moreover, while the current study employed a cross-sectional design, future work could explore longitudinal extensions using Dynamic Bayesian Networks [62,63] to examine temporal changes in emotion regulation and recidivism risk. …” (p.20)

In addition, we have explicitly addressed the ethical considerations related to studying a prison population. The following paragraph was added to clarify the safeguards implemented to ensure voluntary and informed participation:”

“A potential ethical concern related to compensating prisoners is the risk of undue influence, where the compensation might coerce participation. To mitigate this, we took several measures. First, we ensured that the compensation amount of 50,000 won (approximately US $40) was reasonable and not so large as to be coercive. Second, we emphasized that participation was entirely voluntary and that their decision to participate or abstain would neither confer benefits nor impose penalties regarding their future incarceration conditions. Third, we provided comprehensive briefings to ensure that prisoners fully understood their rights and the study's purpose, and that their data would be completely anonymized. These measures ensured that participation was based on informed consent and voluntary choice, minimizing any potential ethical issues related to compensation.” (p.6-7)

5. *Expand on future research directions. What specific questions remain unanswered? How might future studies build on your findings or address the limitations you’ve identified?*

-- For each limitation we mentioned, we discussed the future studies build on our findings as follows:

“First, because all variables were measured through self-report instruments, potential biases such as social desirability and subjective misperception may have influenced participants’ responses. Future research could benefit from incorporating objective indicators—such as physiological measures or behavioral observations—to obtain a more comprehensive assessment of emotion regulation strategies.

Second, although a sample size of more than 500 is substantial given the specialized nature of the offender population—particularly considering the smaller size of certain subgroups such as drug offenders—future studies with larger samples could improve the generalizability of these findings.

Third, our findings are based on a specific population, which may limit the generalizability of the conditional dependence patterns observed among CERS, recidivism, and offense type. … Future studies should therefore include larger and more culturally diverse offender populations to examine the cross-cultural validity of the present findings and test whether the structure of CERS remains stable across contexts.

Fourth, another potential limitation lies in the susceptibility of Bayesian networks to model complexity. … Future research could build on this foundation by incorporating additional variables—such as demographic, psychosocial, or institutional factors—into Bayesian networks with larger samples to more accurately represent the multifaceted nature of offender rehabilitation.

Lastly, while the current study employed a cross-sectional design, future work could explore longitudinal extensions using Dynamic Bayesian Networks [62,63] to examine temporal changes in emotion regulation and recidivism risk.” (pp.19-20)

6. *your discussion back to the gaps in the literature you identified in the introduction. How do your findings address these gaps?*

-- We thank the reviewer for emphasizing the need to connect our findings to the research gaps introduced earlier. In the revision, we have explicitly discussed how the present study addresses those gaps, as follows:

“… This study directly addresses the scarcity of probabilistic, individualized modeling in research on cognitive emotion regulation and recidivism. Whereas previous studies have relied primarily on linear or group-level analyses, our application of Bayesian networks provides a structurally transparent and probabilistically grounded framework that overcomes these limitations. Also, by differentiating adaptive and maladaptive CERS across criminal types and recidivism statuses, the findings reveal complex, non-uniform patterns not captured by conventional methods, while highlighting the importance of accounting for cultural context when modeling emotion-regulation processes in non-Western correctional settings.” (pp.20-21)

7. *End with a strong concluding statement that underscores the significance of your findings and the contribution your study makes to the field.*

-- We appreciate the reviewer’s suggestion. To address this comment, we restructured the Discussion section and added a strong concluding statement that highlights the significance and contributions of our findings. The revised conclusion now reads as follows:

“Despite these limitations, the present study makes a meaningful contribution to correctional psychology by demonstrating the applicability of Bayesian networks in modeling cognitive emotion regulation and recidivism among incarcerated populations. Implementing the Bayesian network developed through the two-stage procedure [38] illuminates the complex relationships between prisoners’ CERS and their likelihood of reoffending through graphical representation. The findings highlight the value of personalized CER rehabilitation programs, aligning with Lipsey and Cullen’s (2007) [31] evidence on the importance of tailored interventions.

This study directly addresses the scarcity of probabilistic, individualized modeling in research on cognitive emotion regulation and recidivism. Whereas previous studies have relied primarily on linear or group-level analyses, our application of Bayesian networks provides a structurally transparent and probabilistically grounded framework that overcomes these limitations. Also, by differentiating adaptive and maladaptive CERS across criminal types and recidivism statuses, the findings reveal complex, non-uniform patterns not captured by conventional methods, while highlighting the importance of accounting for cultural context when modeling emotion-regulation processes in non-Western correctional settings.

Moreover, the ability to simulate individual risk probabilities based on specific offender profiles represents a significant advance in bridging diagnostic insight and intervention planning, addressing a key conceptual and methodological gap in correctional psychology. To promote further application and research, we have made our Bayesian network publicly available at [this link](https://osf.io/7pufw/overview?view_only=44fa3d25788b407d841e286ccf324f9e), enabling clinicians and correctional psychologists to explore the probabilistic relationships between CERS and recidivism across offender types or to develop customized intervention programs aimed at reducing recidivism risk.” (pp 20-21)
