# Supplementary material for: Modeling conditional dependencies between recidivism and cognitive emotion regulation strategies among prisoners using a Bayesian network with interpretable summary indexes
Source: PLoS One. 2026 Aug 6;21(8):e0352880. doi: 10.1371/journal.pone.0352880 (PMC13446662; doi:10.1371/journal.pone.0352880)
Supplement: Peer review history

# Author comment: Author response to Decision Letter 0

**DOI:** 10.1371/journal.pone.0352880.r001

10 Dec 2025

Attachment Submitted filename: Response PLOS_ONE_Letter_v1.docx

# Peer review: Decision Letter 0

**DOI:** 10.1371/journal.pone.0352880.r002

**Authors:** Gerard Hutchinson

25 Mar 2026

PONE-D-25-57503

Modeling conditional dependencies between recidivism and cognitive–emotional regulation strategies among prisoners using a Bayesian network with interpretable summary indexes

PLOS One

Dear Dr. Cho,

Thank you for submitting your manuscript to PLOS ONE. After careful consideration, we feel that it has merit but does not fully meet PLOS ONE’s publication criteria as it currently stands. Therefore, we invite you to submit a revised version of the manuscript that addresses the points raised during the review process.

Please submit your revised manuscript by May 08 2026 11:59PM. If you will need more time than this to complete your revisions, please reply to this message or contact the journal office at plosone@plos.org. When you're ready to submit your revision, log on to https://www.editorialmanager.com/pone/ and select the 'Submissions Needing Revision' folder to locate your manuscript file.

Please include the following items when submitting your revised manuscript:

- A letter that responds to each point raised by the academic editor and reviewer(s). You should upload this letter as a separate file labeled 'Response to Reviewers'.
- A marked-up copy of your manuscript that highlights changes made to the original version. You should upload this as a separate file labeled 'Revised Manuscript with Track Changes'.
- An unmarked version of your revised paper without tracked changes. You should upload this as a separate file labeled 'Manuscript'.

If you would like to make changes to your financial disclosure, please include your updated statement in your cover letter. Guidelines for resubmitting your figure files are available below the reviewer comments at the end of this letter.

If applicable, we recommend that you deposit your laboratory protocols in protocols.io to enhance the reproducibility of your results. Protocols.io assigns your protocol its own identifier (DOI) so that it can be cited independently in the future. For instructions see: https://journals.plos.org/plosone/s/submission-guidelines#loc-laboratory-protocols. Additionally, PLOS ONE offers an option for publishing peer-reviewed Lab Protocol articles, which describe protocols hosted on protocols.io. Read more information on sharing protocols at https://plos.org/protocols?utm_medium=editorial-email&utm_source=authorletters&utm_campaign=protocols.

We look forward to receiving your revised manuscript.

Kind regards,

Gerard Hutchinson, MD

Academic Editor

PLOS One

Journal Requirements:

When submitting your revision, we need you to address these additional requirements.

1.Please ensure that your manuscript meets PLOS ONE's style requirements, including those for file naming. The PLOS ONE style templates can be found at

https://journals.plos.org/plosone/s/file?id=wjVg/PLOSOne_formatting_sample_main_body.pdf and

https://journals.plos.org/plosone/s/file?id=ba62/PLOSOne_formatting_sample_title_authors_affiliations.pdf

2. Please provide additional information regarding the considerations  made for the prisoners included in this study. For instance, please discuss whether participants were able to opt out of the study and whether individuals who did not participate receive the same treatment offered to participants.

3. Thank you for stating the following financial disclosure:

“This research was supported by the National Research Foundation of Korea (NRF) under a grant funded by the Ministry of Science and ICT (MSIT; https://www.msit.go.kr/eng/index.do

) (Grant No. NRF-2019R1F1A1061251). Dr. Youngyoung Choi was the recipient of this grant.

Open-access publication fees for this article were supported by The Ohio State University’s Open Access Publishing Fund.”

Please state what role the funders took in the study.  If the funders had no role, please state: "The funders had no role in study design, data collection and analysis, decision to publish, or preparation of the manuscript."

If this statement is not correct you must amend it as needed.

Please include this amended Role of Funder statement in your cover letter; we will change the online submission form on your behalf.

4. We note that you have indicated that there are restrictions to data sharing for this study. PLOS only allows data to be available upon request if there are legal or ethical restrictions on sharing data publicly. For more information on unacceptable data access restrictions, please see http://journals.plos.org/plosone/s/data-availability#loc-unacceptable-data-access-restrictions.

Before we proceed with your manuscript, please address the following prompts:

a) If there are ethical or legal restrictions on sharing a de-identified data set, please explain them in detail (e.g., data contain potentially identifying or sensitive patient information, data are owned by a third-party organization, etc.) and who has imposed them (e.g., a Research Ethics Committee or Institutional Review Board, etc.). Please also provide contact information for a data access committee, ethics committee, or other institutional body to which data requests may be sent.

b) If there are no restrictions, please upload the minimal anonymized data set necessary to replicate your study findings to a stable, public repository and provide us with the relevant URLs, DOIs, or accession numbers. For a list of recommended repositories, please see

https://journals.plos.org/plosone/s/recommended-repositories. You also have the option of uploading the data as Supporting Information files, but we would recommend depositing data directly to a data repository if possible.

We will update your Data Availability statement on your behalf to reflect the information you provide.

5. If the reviewer comments include a recommendation to cite specific previously published works, please review and evaluate these publications to determine whether they are relevant and should be cited. There is no requirement to cite these works unless the editor has indicated otherwise.

6. Please review your reference list to ensure that it is complete and correct. If you have cited papers that have been retracted, please include the rationale for doing so in the manuscript text, or remove these references and replace them with relevant current references. Any changes to the reference list should be mentioned in the rebuttal letter that accompanies your revised manuscript. If you need to cite a retracted article, indicate the article’s retracted status in the References list and also include a citation and full reference for the retraction notice.

[Note: HTML markup is below. Please do not edit.]

Reviewers' comments:

Reviewer's Responses to Questions

**Comments to the Author**

1. Is the manuscript technically sound, and do the data support the conclusions?

The manuscript must describe a technically sound piece of scientific research with data that supports the conclusions. Experiments must have been conducted rigorously, with appropriate controls, replication, and sample sizes. The conclusions must be drawn appropriately based on the data presented.

Reviewer #1: Yes

Reviewer #2: Yes

**********

2. Has the statistical analysis been performed appropriately and rigorously?

Reviewer #1: Yes

Reviewer #2: Yes

**********

3. Have the authors made all data underlying the findings in their manuscript fully available?

The PLOS Data policy requires authors to make all data underlying the findings described in their manuscript fully available without restriction, with rare exception (please refer to the Data Availability Statement in the manuscript PDF file). The data should be provided as part of the manuscript or its supporting information, or deposited to a public repository. For example, in addition to summary statistics, the data points behind means, medians and variance measures should be available. If there are restrictions on publicly sharing data—e.g. participant privacy or use of data from a third party—those must be specified.

Reviewer #1: Yes

Reviewer #2: No

**********

4. Is the manuscript presented in an intelligible fashion and written in standard English?

PLOS ONE does not copyedit accepted manuscripts, so the language in submitted articles must be clear, correct, and unambiguous. Any typographical or grammatical errors should be corrected at revision, so please note any specific errors here.

Reviewer #1: Yes

Reviewer #2: Yes

**********

5. Review Comments to the Author

Please use the space provided to explain your answers to the questions above. You may also include additional comments for the author, including concerns about dual publication, research ethics, or publication ethics. (Please upload your review as an attachment if it exceeds 20,000 characters)

Reviewer #1: The manuscript makes a valuable contribution to correctional psychology by demonstrating how Bayesian networks can model complex, probabilistic relationships between emotion regulation and recidivism in ways that conventional statistical methods cannot. The findings have both theoretical implications for understanding offense-specific emotion regulation patterns and practical applications for personalized intervention planning. The authors have thoroughly addressed previous reviewer concerns, and the manuscript is suitable for publication in PLOS ONE.

Reviewer #2: PEER REVIEW REPORT

Modeling conditional dependencies between recidivism and cognitive–emotion regulation strategies among prisoners using a Bayesian network with interpretable summary indexes

Major Concerns

Discretization of GSCA Component Scores Not Described

Netica requires discrete nodes, yet the continuous GSCA component scores from Stage 1 are entered into the BN without any description of how they were discretized. The Methods section must specify: (1) the discretization method (equal-frequency, equal-width, percentile, or substantive thresholds); and (2) the number of discrete states per node. The Results section refers to "higher," "medium," and "lower" levels (p. 22), implying a three-state scheme, but this is never stated or justified.

The Discussion should also acknowledge that discretization entails information loss and that results are sensitive to the choice of cut-points. Future research directions worth noting include sensitivity analyses comparing alternative discretization schemes, or the use of continuous BN extensions (e.g., conditional Gaussian models) that eliminate the need for discretization entirely.

Model Evaluation: AUC Computed on Training Data

The reported AUC of .763 appears to be evaluated on the same 500-case dataset used for parameter estimation, producing an optimistically biased performance estimate — a concern compounded by the relatively small sample and the number of nodes in the network. The authors should replace this in-sample AUC with a proper out-of-sample estimate via k-fold cross-validation (e.g., 10-fold, reporting mean AUC with confidence intervals), a held-out test set, or bootstrapped bias-corrected estimation. The current claim that the network "outperforms random guessing" is insufficiently supported without one of these approaches.

Critical Citation Error: Korean CERQ Reference

In the Measures section (p. 8), the authors write: “Cognitive-emotion regulation strategies (CERS) were assessed using the Korean version of Cognitive Emotion Regulation Questionnaires (CERQ), validated by Ahn, Lee, and Joo (2013) [43].” However, Reference [43] in the reference list is:

Pavlenko T, Rosen D von. Bayesian network classifiers in a high dimensional framework. AI '02… (2002).

The correct reference for Ahn, Lee, and Joo (2013) appears to be [47] in the reference list. This is a substantive citation error that misattributes the validation of the primary measurement instrument. The authors must verify that all in-text citation numbers match their reference list entries.

Drug Offender Subgroup Too Small for Reliable Inference

The drug-related crimes subgroup comprises only 20 participants (4.0% of the sample). Despite this, Figure 6 and the associated Discussion present specific conditional probability estimates for drug offenders to two decimal places (e.g., “91.8% vs. 74.7%” for adaptive strategy use). Conditional probability tables for a subgroup of n=20, further divided by recidivism status, are estimated from single-digit cell counts, rendering these estimates highly unstable and their interpretation unreliable.

The authors must either: (a) acknowledge this as a critical limitation and refrain from drawing substantive conclusions from the drug-offender analysis; or (b) provide bootstrapped confidence intervals for all conditional probabilities reported in Figures 5–7 to reflect the uncertainty associated with small cell sizes. Presenting point estimates without uncertainty quantification for such small subgroups risks misleading readers.

Minor Concerns

Descriptive Statistics Inconsistency (Text vs. Table 1)

The manuscript text (p. 8) reports the mean number of incarcerations as 2.39 (SD = 2.20), but Table 1 reports the same variable as Mean = 2.98, SD = 2.63. These values are irreconcilable. The authors must verify which figure is correct and ensure consistency between the text and the table. Additionally, the variable “Age of first conviction” (Mean = 30.22, SD = 12.81) appears in Table 1 but is not mentioned in the text; it should either be described or removed from the table.

Disposition of “Others” Offense Category

Table 1 includes an “Others” offense category (n = 18, 3.6%). It is unclear whether these participants were included in or excluded from the Bayesian network analysis, which models five specific crime types (homicide, violent offenses, sexual violence, property, drug). If excluded, the effective analysis sample would be n = 482, not n = 500. This must be clarified.

Uncertainty Propagation Across Stages

The two-stage procedure treats GSCA-derived component scores as fixed observed values in the BN, thereby ignoring the estimation uncertainty inherent in Stage 1. In particular, the standard errors and confidence intervals reported in Table 2 for GSCA weight and loading estimates are not carried forward to Stage 2. This leads to underestimation of the uncertainty surrounding the conditional probability estimates produced by the BN. The authors should at minimum acknowledge this limitation and, ideally, consider a sensitivity analysis or bootstrapped approach that captures the combined uncertainty across both stages.

Typographical Errors

The running head on page 1 reads “MODELING CONDITONAL DEPENENCY B/W RECIDIVISM AND CERS,” which contains two spelling errors (“CONDITONAL” and “DEPENENCY”) and an informal abbreviation (“B/W”). The full running head on subsequent pages retains the spelling errors. These should be corrected.

**********

6. PLOS authors have the option to publish the peer review history of their article (what does this mean?). If published, this will include your full peer review and any attached files.

If you choose “no”, your identity will remain anonymous but your review may still be made public.

**Do you want your identity to be public for this peer review?**  For information about this choice, including consent withdrawal, please see our Privacy Policy.

Reviewer #1: No

Reviewer #2: No

**********

[NOTE: If reviewer comments were submitted as an attachment file, they will be attached to this email and accessible via the submission site. Please log into your account, locate the manuscript record, and check for the action link "View Attachments". If this link does not appear, there are no attachment files.]

To ensure your figures meet our technical requirements, please review our figure guidelines: https://journals.plos.org/plosone/s/figures

You may also use PLOS’s free figure tool, NAAS, to help you prepare publication quality figures: https://journals.plos.org/plosone/s/figures#loc-tools-for-figure-preparation.

NAAS will assess whether your figures meet our technical requirements by comparing each figure against our figure specifications.

Attachment Submitted filename: review_report_PONE_Oh.docx

# Author comment: Author response to Decision Letter 1

**DOI:** 10.1371/journal.pone.0352880.r003

22 May 2026

PONE-D-25-57503: Modeling conditional dependencies between recidivism and cognitive emotion regulation strategies among prisoners using a Bayesian network with interpretable summary indexes

We thank the Editor and two anonymous reviewers for their thoughtful and constructive comments. For their convenience, we repeat every comment in italics and then respond to the comment in regular font.

Response to Reviewer #1

1. The manuscript makes a valuable contribution to correctional psychology by demonstrating how Bayesian networks can model complex, probabilistic relationships between emotion regulation and recidivism in ways that conventional statistical methods cannot. The findings have both theoretical implications for understanding offense-specific emotion regulation patterns and practical applications for personalized intervention planning. The authors have thoroughly addressed previous reviewer concerns, and the manuscript is suitable for publication in PLOS ONE. The authors have thoroughly addressed previous reviewer concerns, and the manuscript is suitable for publication in PLOS ONE.

-- We sincerely appreciate the reviewer’s positive assessment of our manuscript and the thoughtful comments they provided in the previous review. We made substantial efforts to address their concerns, and we are very pleased to hear that the reviewer appreciate both our revisions and the manuscript’s contribution.

Response to Reviewer #2

Major comments

1. [Discretization of GSCA Component Scores Not Described] Netica requires discrete nodes, yet the continuous GSCA component scores from Stage 1 are entered into the BN without any description of how they were discretized. The Methods section must specify: (1) the discretization method (equal-frequency, equal-width, percentile, or substantive thresholds); and (2) the number of discrete states per node. The Results section refers to "higher," "medium," and "lower" levels (p. 22), implying a three-state scheme, but this is never stated or justified. The Discussion should also acknowledge that discretization entails information loss and that results are sensitive to the choice of cut-points. Future research directions worth noting include sensitivity analyses comparing alternative discretization schemes, or the use of continuous BN extensions (e.g., conditional Gaussian models) that eliminate the need for discretization entirely.

-- We appreciate the reviewer’s helpful comment. Although the Bayesian network file included the discrete states and cut-points, we agree that the discretization procedure should have been described and justified more explicitly in the manuscript. Accordingly, we revised the Methods and Results sections to specify the discretization procedure for the CERS component scores and its rationale.

In the Methods section, we added the following clarification:

“After selecting the better-fitting GSCA model in Stage 1, we used the resulting components as nodes in the Bayesian network [32]. Their scores were then used to estimate the Bayesian network parameters in Stage 2. Because a discrete Bayesian network requires categorical node states, the GSCA-derived CERS component scores were discretized into three theoretically interpretable frequency-based states before parameter estimation; the specific cutoffs are reported with the Bayesian network results.” (p. 12)

In the Results section, we further specified the number of states, the cutoffs, and the rationale for using substantive thresholds:

“Figure 2 depicts the Bayesian network constructed with first- and second-order CERS components derived from Stage 1. The first-order components correspond to nine CERS, while the second-order components comprise one adaptive strategy and two maladaptive strategies. Since the second maladaptive strategy is identical to the other-blame strategy, we included only a single node (i.e., box) to represent both components. Each first- and second-order CERS component was discretized into three states using the following cutoffs: 1–2.5 = ‘Rarely or less,’ 2.5–3.5 = ‘Occasionally,’ and 3.5–5 = ‘Frequently or more.’ This discretization was possible because the component scores for the CERQ strategies obtained from Convex GSCA can be interpreted in relation to the original five-point response scale, ranging from 1 (“Almost Never”) to 5 (“Almost Always”) [35]. This allowed us to use theoretically interpretable substantive thresholds rather than purely distribution-based cut-points, such as tertiles based on the sample distribution.” (p. 15)

We also revised the terminology to use the frequency-based state labels consistently throughout the manuscript. In addition, we updated the Discussion section to acknowledge that discretization may involve some loss of information and that the Bayesian network results can be sensitive to the number of states and selected cut-points. We added the following limitation and future research direction:

“… However, as reflected in the modest LOOCV-AUC value, the model’s predictive generalizability remained limited, and the resulting conditional probability patterns may be sensitive to the number of states and the selected thresholds. Alternative discretization schemes, a larger number of states, or more data-driven cut-points may yield different conditional probability patterns and potentially improve predictive generalizability. Future research should therefore examine the robustness of the BN results across different state definitions, thresholding strategies, and model structures using larger samples.” (p. 22)

Lastly, we appreciate the reviewer’s suggestion regarding continuous Bayesian network extensions. Nonetheless, we retained the discrete Bayesian network framework because it provides readily interpretable conditional probability tables that are well aligned with the substantive and applied goals of the study. Accordingly, we clarified in the Methods section why the conventional discrete Bayesian network framework was appropriate for the present study:

“We used a discrete Bayesian network, in which each node is represented by a finite set of states and the dependencies among nodes are summarized through conditional probability tables. Although Bayesian network extensions can accommodate continuous variables, we proceeded with the traditional discrete Bayesian network framework because the substantive aim of the study was to model interpretable categorical patterns of cognitive emotion regulation and recidivism across crime types. In correctional assessment and intervention planning, decision-making often involves categorical judgments, such as identifying whether a risk-related characteristic is low or high, or whether a specific intervention target is more or less salient for a given offender group. A discrete Bayesian network is therefore well aligned with the present goal of representing conditional dependency patterns in a form that can be directly inspected and interpreted.” (p. 10)

2. [Model Evaluation: AUC Computed on Training Data] The reported AUC of .763 appears to be evaluated on the same 500-case dataset used for parameter estimation, producing an optimistically biased performance estimate — a concern compounded by the relatively small sample and the number of nodes in the network. The authors should replace this in-sample AUC with a proper out-of-sample estimate via k-fold cross-validation (e.g., 10-fold, reporting mean AUC with confidence intervals), a held-out test set, or bootstrapped bias-corrected estimation. The current claim that the network "outperforms random guessing" is insufficiently supported without one of these approaches.

-- We appreciate the reviewer’s important comment. We agree that the AUC reported in the previous version was computed on the same data used for parameter estimation and therefore should not be interpreted as an out-of-sample estimate of predictive performance. We also agree that the previous statement that the network “outperforms random guessing” could be read as overstating the predictive performance of the model, and we have removed this statement from the revised manuscript.

At the same time, we would like to clarify that the primary aim of the present study was not to develop or validate a high-accuracy recidivism prediction model. As reflected in the title and abstract, the purpose of the study was to model conditional dependencies between recidivism and cognitive emotion regulation strategies among prisoners, conditioning on crime type, using a Bayesian network with interpretable summary indexes. Thus, the originally reported in-sample AUC was intended as a descriptive index of the fitted network’s apparent discriminative performance within the analytic sample, rather than as evidence of predictive generalizability.

To address the reviewer’s concern, we first updated the Methods section to describe the two AUC values and their interpretations as follows:

“... We then evaluated the trained network using both an in-sample AUC and a cross-validated AUC. The AUC summarizes the discriminative ability of the network, with .5 corresponding to chance-level discrimination and larger values indicating greater discrimination [44]. The in-sample AUC was treated as a descriptive index of apparent discriminative performance within the analytic sample, whereas the cross-validated AUC was used to examine the extent to which this discriminative pattern was retained for held-out cases.” (p. 13)

We additionally computed a leave-one-out cross-validated AUC and reported its value in the Results section. Because the Bayesian network structure was fixed rather than learned from the data, each leave-one-out iteration re-estimated the conditional probability tables using the remaining N-1cases and then obtained the predicted probability of recidivism for the held-out case. The final LOOCV-AUC was computed from the held-out predicted probabilities across all cases.

The resulting LOOCV-AUC was 0.552, indicating limited out-of-sample discriminative performance and a substantial reduction from the in-sample AUC. Accordingly, we revised the Results and Discussion sections to clarify the limited predictive generalizability of the fitted Bayesian network and to reposition the model as an exploratory and interpretable representation of conditional dependency patterns, rather than as a validated high-accuracy prediction model. For example, in the Results section, we now state:

“... To examine the extent to which this discriminative pattern was retained for held-out cases, we additionally computed a leave-one-out cross-validated AUC (LOOCV-AUC). The LOOCV-AUC was .552, indicating limited out-of-sample discriminative performance. Thus, the fitted Bayesian network was used primarily to examine conditional probability patterns linking prisoners’ CERS components, crime type, and recidivism, rather than as a validated high-accuracy prediction model.” (p. 16)

We also revised the interpretation of subgroup- and profile-level posterior probabilities throughout the manuscript to avoid implying validated individualized prediction. In particular, we now summarize the Results section as follows:

“Taken together, these results suggest that the Bayesian network provided an interpretable and descriptive representation of conditional dependencies between CERS components and recidivism across crime types in the analytic sample. However, given the modest cross-validated AUC and the small size of some crime-type subgroups, particularly drug offenders, the posterior probability patterns for subgroups and specified offender profiles should be interpreted cautiously and should not be taken as evidence of validated predictive performance or individualized risk prediction.” (p. 18)

Finally, we explicitly acknowledged this issue as a limitation in the Discussion section. Specifically, we added the following statement:

“... In addition, because the discrete Bayesian network involved multiple parent-state configurations, some conditional probability estimates may have been based on sparse cells. This issue was also reflected in the modest leave-one-out cross-validated AUC, suggesting that the predictive generalizability of the current network should be interpreted cautiously. Future studies with larger and more balanced samples across crime types are needed to evaluate the stability and generalizability of the observed CERS–recidivism dependency patterns.” (p. 21)

3. [Critical Citation Error: Korean CERQ Reference] In the Measures section (p. 8), the authors write: “Cognitive-emotion regulation strategies (CERS) were assessed using the Korean version of Cognitive Emotion Regulation Questionnaires (CERQ), validated by Ahn, Lee, and Joo (2013) [43].” However, Reference [43] in the reference list is:

Pavlenko T, Rosen D von. Bayesian network classifiers in a high dimensional framework. AI '02… (2002).

The correct reference for Ahn, Lee, and Joo (2013) appears to be [47] in the reference list. This is a substantive citation error that misattributes the validation of the primary measurement instrument. The authors must verify that all in-text citation numbers match their reference list entries.

-- We thank the reviewer for catching this important citation error. The error appears to have occurred when the reference numbering changed during a previous revision of the manuscript. We corrected the Measures section so that the Korean validation of the CERQ is now cited as Ahn, Lee, and Joo (2013) [30]. We also clarified the original CERQ references by citing Garnefski, Kraaij, and Spinhoven (2001) [11] for the original development of the CERQ and Garnefski and Kraaij (2007) [12] for further psychometric evidence.

In addition, we carefully checked the in-text citations and reference list throughout the manuscript and corrected the mismatches we identified. We appreciate the reviewer’s careful reading and helpful attention to this issue.

4. [Drug Offender Subgroup Too Small for Reliable Inference] The drug-related crimes subgroup comprises only 20 participants (4.0% of the sample). Despite this, Figure 6 and the associated Discussion present specific conditional probability estimates for drug offenders to two decimal places (e.g., “91.8% vs. 74.7%” for adaptive strategy use). Conditional probability tables for a subgroup of n=20, further divided by recidivism status, are estimated from single-digit cell counts, rendering these estimates highly unstable and their interpretation unreliable.

The authors must either: (a) acknowledge this as a critical limitation and refrain from drawing substantive conclusions from the drug-offender analysis; or (b) provide bootstrapped confidence intervals for all conditional probabilities reported in Figures 5–7 to reflect the uncertainty associated with small cell sizes. Presenting point estimates without uncertainty quantification for such small subgroups risks misleading readers.

-- We appreciate the reviewer’s important comment. We agree that the drug-related crime subgroup was small and that conditional probability estimates for this subgroup may be unstable, especially when further conditioned on recidivism status and CERS component levels. Because correctional datasets of this kind are difficult to obtain, our intention in retaining the drug-related crime category was to provide a descriptive account of the crime types represented in the available sample, not to draw strong subgroup-specific substantive or clinical conclusions. In the revised manuscript, we therefore rewrote the relevant Results and Discussion passages so that the drug-offender findings are presented as sample-based, descriptive, and exploratory. For example, we now summarize the findings as follows:

“Taken together, these results suggest that the Bayesian network provided an interpretable and descriptive representation of conditional dependencies between CERS components and recidivism across crime types in the analytic sample. However, given the modest cross-validated AUC and the small size of some crime-type subgroups, particularly drug offenders, the posterior probability patterns for subgroups and specified offender profiles should be interpreted cautiously and should not be taken as evidence of validated predictive performance or individualized risk

Attachment Submitted filename: Response_Letter_ PLOS_ONE_R1_v2.docx

# Peer review: Decision Letter 1

**DOI:** 10.1371/journal.pone.0352880.r004

**Authors:** Gerard Hutchinson

16 Jun 2026

Modeling conditional dependencies between recidivism and cognitive emotion regulation strategies among prisoners using a Bayesian network with interpretable summary indexes

PONE-D-25-57503R1

Dear Dr. Cho,

We’re pleased to inform you that your manuscript has been judged scientifically suitable for publication and will be formally accepted for publication once it meets all outstanding technical requirements.

Within one week, you’ll receive an e-mail detailing the required amendments. When these have been addressed, you’ll receive a formal acceptance letter and your manuscript will be scheduled for publication.

An invoice will be generated when your article is formally accepted. Please note, if your institution has a publishing partnership with PLOS and your article meets the relevant criteria, all or part of your publication costs will be covered. Please make sure your user information is up-to-date by logging into Editorial Manager at Editorial Manager® and clicking the ‘Update My Information' link at the top of the page. For questions related to billing, please contact billing support.

If your institution or institutions have a press office, please notify them about your upcoming paper to help maximize its impact. If they’ll be preparing press materials, please inform our press team as soon as possible -- no later than 48 hours after receiving the formal acceptance. Your manuscript will remain under strict press embargo until 2 pm Eastern Time on the date of publication. For more information, please contact onepress@plos.org.

Kind regards,

Gerard Hutchinson, MD

Academic Editor

PLOS One

Additional Editor Comments (optional):

Reviewers' comments:

Reviewer's Responses to Questions

**Comments to the Author**

1. If the authors have adequately addressed your comments raised in a previous round of review and you feel that this manuscript is now acceptable for publication, you may indicate that here to bypass the “Comments to the Author” section, enter your conflict of interest statement in the “Confidential to Editor” section, and submit your "Accept" recommendation.

Reviewer #2: All comments have been addressed

**********

2. Is the manuscript technically sound, and do the data support the conclusions?

The manuscript must describe a technically sound piece of scientific research with data that supports the conclusions. Experiments must have been conducted rigorously, with appropriate controls, replication, and sample sizes. The conclusions must be drawn appropriately based on the data presented.

Reviewer #2: Yes

**********

3. Has the statistical analysis been performed appropriately and rigorously?

Reviewer #2: Yes

**********

4. Have the authors made all data underlying the findings in their manuscript fully available?

The PLOS Data policy requires authors to make all data underlying the findings described in their manuscript fully available without restriction, with rare exception (please refer to the Data Availability Statement in the manuscript PDF file). The data should be provided as part of the manuscript or its supporting information, or deposited to a public repository. For example, in addition to summary statistics, the data points behind means, medians and variance measures should be available. If there are restrictions on publicly sharing data—e.g. participant privacy or use of data from a third party—those must be specified.

Reviewer #2: No

**********

5. Is the manuscript presented in an intelligible fashion and written in standard English?

PLOS ONE does not copyedit accepted manuscripts, so the language in submitted articles must be clear, correct, and unambiguous. Any typographical or grammatical errors should be corrected at revision, so please note any specific errors here.

Reviewer #2: Yes

**********

6. Review Comments to the Author

Please use the space provided to explain your answers to the questions above. You may also include additional comments for the author, including concerns about dual publication, research ethics, or publication ethics. (Please upload your review as an attachment if it exceeds 20,000 characters)

Reviewer #2: All of my previous comments have been adequately addressed in the revised manuscript. I have no further concerns and recommend acceptance.

**********

7. PLOS authors have the option to publish the peer review history of their article (what does this mean?). If published, this will include your full peer review and any attached files.

If you choose “no”, your identity will remain anonymous but your review may still be made public.

**Do you want your identity to be public for this peer review?**  For information about this choice, including consent withdrawal, please see our Privacy Policy.

Reviewer #2: No

**********

# Editor report: Acceptance letter

**DOI:** 10.1371/journal.pone.0352880.r005

**Authors:** Gerard Hutchinson

PONE-D-25-57503R1

PLOS One

Dear Dr. Cho,

I'm pleased to inform you that your manuscript has been deemed suitable for publication in PLOS One. Congratulations! Your manuscript is now being handed over to our production team.

At this stage, our production department will prepare your paper for publication. This includes ensuring the following:

* All references, tables, and figures are properly cited

* All relevant supporting information is included in the manuscript submission,

* There are no issues that prevent the paper from being properly typeset

You will receive further instructions from the production team, including instructions on how to review your proof when it is ready. Please keep in mind that we are working through a large volume of accepted articles, so please give us a few days to review your paper and let you know the next and final steps.

Lastly, if your institution or institutions have a press office, please let them know about your upcoming paper now to help maximize its impact. If they'll be preparing press materials, please inform our press team within the next 48 hours. Your manuscript will remain under strict press embargo until 2 pm Eastern Time on the date of publication. For more information, please contact onepress@plos.org.

You will receive an invoice from PLOS for your publication fee after your manuscript has reached the completed accept phase. If you receive an email requesting payment before acceptance or for any other service, this may be a phishing scheme. Learn how to identify phishing emails and protect your accounts at https://explore.plos.org/phishing.

If we can help with anything else, please email us at customercare@plos.org.

Thank you for submitting your work to PLOS ONE and supporting open access.

Kind regards,

PLOS ONE Editorial Office Staff

on behalf of

Dr. Gerard Hutchinson

Academic Editor

PLOS One
